# Consecutive Positive Feedback Loops Create a Bistable Switch that Controls Preadipocyte-to-Adipocyte Conversion

**DOI:** 10.1016/j.celrep.2012.08.038

**Published:** 2012-10-11

**Authors:** Byung Ouk Park, Robert Ahrends, Mary N. Teruel

**Affiliations:** 1 Department of Chemical and Systems Biology, Stanford University, Stanford, CA 94305, USA

## Abstract

Adipogenesis, or the conversion of proliferating preadipocytes into nondividing adipocytes, is an important part of the vertebrate weight-maintenance program. It is not yet understood how and when an irreversible transition occurs into a distinct state capable of accumulating lipid. Here, we use single-cell fluorescence imaging to show that an all-or-none switch is induced before lipid accumulation occurs. Conversion begins by glucocorticoid and cAMP signals raising C/EBPβ levels above a critical threshold, triggering three consecutive positive feedback loops: from PPARγ to C/EBPα, then to C/EBPβ, and last to the insulin receptor. Experiments and modeling show that these feedbacks create a robust, irreversible transition to a terminally differentiated state by rejecting short- and low-amplitude stimuli. After the differentiation switch is triggered, insulin controls fat accumulation in a graded fashion. Altogether, our study introduces a regulatory motif that locks cells in a differentiated state by engaging a sequence of positive feedback loops.

## INTRODUCTION

Adipocytes, or fat cells, are essential for human health, carrying out critical functions including cushioning and insulating the body and internal organs, storing up to 80%–90% of the body's energy, and regulating glucose homeostasis and energy metabolism by secreting key hormones such as leptin, adiponectin, and TNF-α (Ahima and Flier, 2000; Rosen and Spiegelman, 2006). With the current epidemic of obesity and the strong correlations of obesity with diabetes, cardiovascular disease, and cancer, understanding the molecular mechanisms underlying adipogenesis, or the conversion of dividing preadipocytes into nondividing, lipid-accumulating fat cells, is of great scientific and medical interest. Many regulatory factors have been implicated in adipogenesis and have been depicted in summary diagrams (i.e., Cristancho and Lazar, 2011; Farmer, 2006; Lowe et al., 2011). However, static diagrams are inadequate for understanding the key steps in a dynamic and complex process like adipogenesis. In addition, to understand if, when, and how a clear commitment decision is made and a cell transitions irreversibly from a distinct preadipocyte state into a distinct adipocyte state requires measurements at the single-cell level. Such a single-cell analysis has not yet been performed during adipogenesis.

Adipogenesis occurs over several days and can be triggered by a number of hormonal stimuli. Several cell models have been established to study adipogenesis in vitro (Green and Kehinde, 1976; Wolins et al., 2006). In these models, adipogenesis is induced by the addition of glucocorticoid and insulin together with different strategies to increase cAMP. Key players in the transcriptional network controlling adipogenesis include the transcription factors C/EBPβ and C/EBPα and the nuclear receptor PPARγ, which is often described as a master regulator because it has been shown to be both necessary and sufficient for fat cell differentiation (Tontonoz and Spiegelman, 2008). Expression of C/EBPβ has been shown to induce the expression of PPARγ (Wu et al., 1996), most likely due to direct regulation because C/EBP binding sites have been identified in the PPARγ promoter (Zhu et al., 1995; Fajas et al., 1997). Previous work showed that a positive feedback exists between PPARγ and C/EBPα, and it has been suggested that this positive feedback is important to induce a terminal differentiated state (El-Jack et al., 1999; Rosen et al., 2002; Wu et al., 1999). However, positive feedbacks in cell regulatory systems are common, and their function in many cases is simply to amplify a transmitted signal (Brandman and Meyer, 2008). To trigger an irreversible decision or bistable switch, a cooperative regulatory step is required in addition to positive feedback. Also, in most known biological switch mechanisms such as the oscillations in Ca^2+^ signaling and the G2/M cell-cycle decision, cells rely on more than one positive feedback (Brandman et al., 2005). Neither multiple positive feedbacks nor cooperativity in the activation steps has been described in adipocyte differentiation.

Even the presence of multiple, cooperative positive feedbacks does not prove that a bistable, irreversible, or differentiated state is induced; this depends further on the specific enzymatic parameters. To show that a bimodal state is induced, single-cell experimental data using markers for the feedback regulators are first needed. To then prove that such a bimodal state is irreversible or bistable, one has to show that the inducing signals can be lowered or removed without losing the new differentiated state created by these feedback regulators (Pomerening et al., 2003; Yao et al., 2008). Finally, it has not yet been determined whether the induction of a bistable differentiation switch occurs independently of fat accumulation because current protocols typically use lipid accumulation as the marker for the differentiated state. These considerations provided the incentive for the studies we pursued here to uncover the molecular mechanisms triggering a potential irreversible bistable switch, and also to determine whether the triggering of such a switch precedes, coincides, or involves lipid synthesis.

To achieve this goal, we developed and applied an image-based approach to simultaneously quantify multiple key parameters in thousands of single cells over the time course of adipogenesis. Our analysis showed that the fat cell differentiation process is bimodal and that a clear decision is made early in differentiation before lipogenesis occurs. We identified a reinforcing feedback loop from PPARγ back to C/EBPβ that engages with a marked delay after a first positive feedback between PPARγ and C/EBPα. This delay is caused by a requirement for higher PPARγ activity for the second feedback to be triggered. We then identified a third commitment step, in which PPARγ expression is further boosted by a positive feedback between PPARγ and the insulin receptor that again engages with a delay after the first two positive feedbacks. We used these single-cell measurements to generate a quantitative model of the differentiation decision. Together with experimental data, model analysis showed that this consecutive feedback loop design is uniquely suited to lock cells in a differentiated state. Thus, our study introduces a regulatory design whereby multiple positive feedback loops sequentially engage with time delays to generate a robust transition to a terminally differentiated state.

## RESULTS

### Single-Cell Analysis of Adipogenic Transcription Factor Expression and Lipid Droplet Formation

To identify a potential bistable switch in the adipocyte differentiation path, we developed a multiparameter, single-cell assay to measure expression of key transcription factors and lipid droplet content over the time course of adipogenesis in both 3T3-L1 cells, a mouse embryo-derived cultured adipocyte model (Green and Kehinde, 1976), as well as OP9 cells, a bone marrow-derived adipocyte model. We and others have verified that OP9 and 3T3-L1 cells have similar adipocyte differentiation characteristics, although OP9 cells do differentiate faster than 3T3-L1 cells (Figures S1A and S1B; Wolins et al., 2006). OP9 cells represent late-stage preadipocytes and thus are more advanced in the differentiation process to become adipocytes (Wolins et al., 2006).

**Figure 1:**
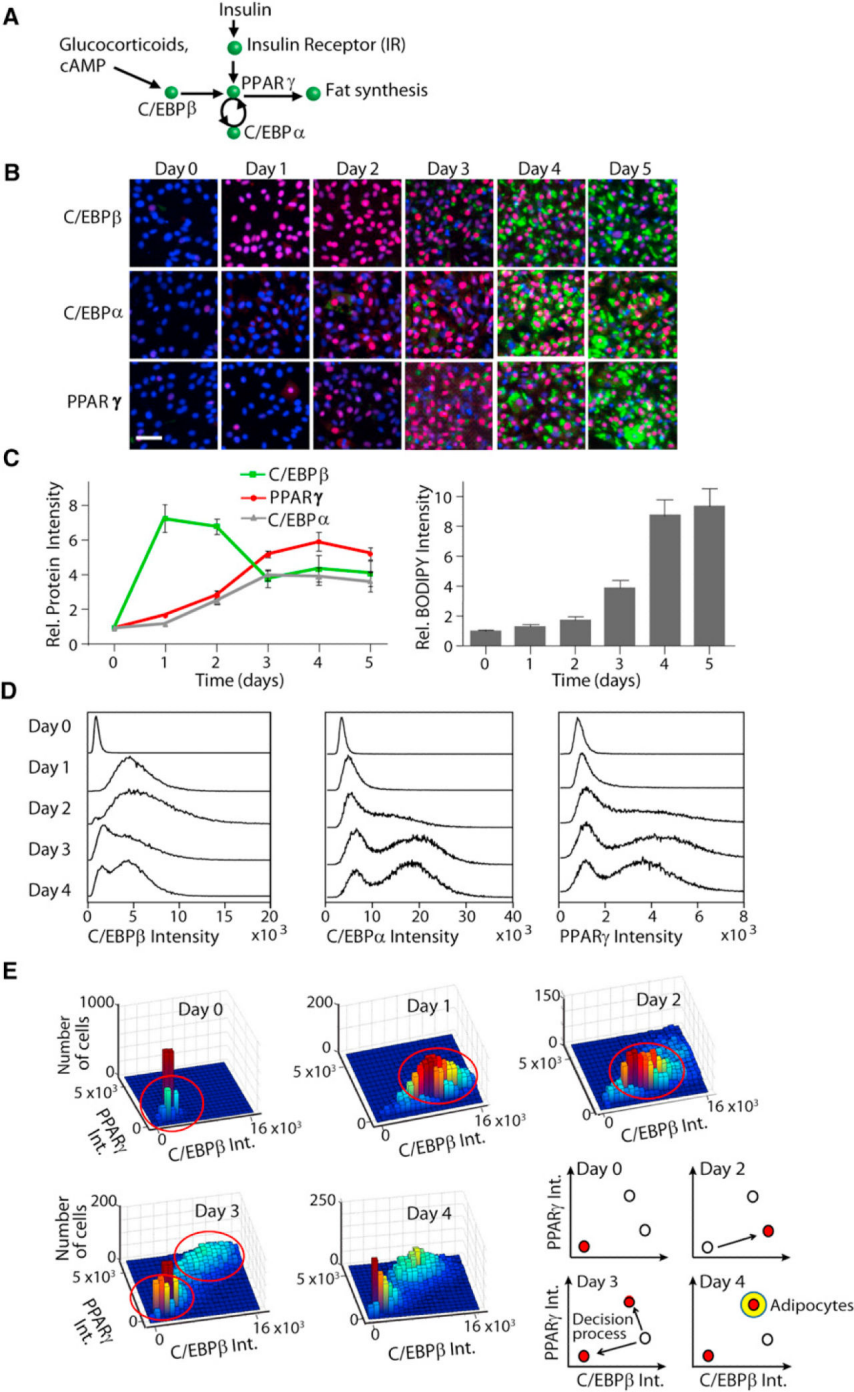
Testing for the Existence of Distinct Cell Differentiation States (A) Current model of adipogenesis is shown. (B) Development of a single-cell approach to measure expression of key transcription factors and lipid accumulation over the time course of adipogenesis is illustrated. Immunohistochemistry staining of OP9 cells using specific antibodies to visualize PPARγ, C/EBPα, and C/EBPβ (red), BODIPY 493/503 to visualize lipid droplets (green), and Hoechst to visualize nuclei (blue) is presented. Scale bar, 40 μm. (C) PPARγ, C/EBPα, and C/EBPβ concentrations were obtained by averaging intensities of antibody staining from the nuclei of individual cells (right). Total cellular lipid droplet content was obtained by averaging BODIPY intensities from the cytosol of individual cells (left). Approximately 25,000 cells were used for each time point. Error bars show SE calculated from three independent experiments. All values are normalized to the respective average day 0 (unstimulated) values. Rel. Protein Intensity, relative protein intensity; Rel. BODIPY Intensity, relative BODIPY intensity. (D) Histograms show number of cells (y axis) with the specified concentrations of PPARγ, C/EBPα, or C/EBPβ (x axis). Approximately 25,000 cells were used for each histogram. (E) 3D histograms show number of cells (z axis) with the specified relative nuclear concentrations of C/EBPβ (x axis) and PPARγ (y axis). Approximately 7,000 cells were used for each histogram. Right bottom shows a schematic of the decision process. Int., intensity. See also Figure S1.

Figure 1A shows a schematic representation of transcriptional regulators that have been shown to control adipogenesis. C/EBPβ expression is upregulated by glucocorticoid and cAMP (Yeh et al., 1995), and PPARγ expression by C/EBPβ, C/EBPα, and insulin (Kim et al., 1998), with PPARγ then driving adipogenesis. Adipogenesis is commonly induced by growing preadipocyte cells such as OP9 or 3T3-L1 cells to confluency and then applying an adipogenic mixture consisting of insulin, fetal bovine serum (FBS), dexamethasone (dex), which is a synthetic glucocorticoid, and 3-isobutyl-1-methylxanthine (IBMX), which is an inhibitor of phosphodiesterase that increases cAMP levels. After 2 days, the glucocorticoid and cAMP stimuli are removed, and the media are replaced with media containing only insulin and FBS.

We performed single-cell image analysis of adipogenesis in both OP9 and 3T3-L1 cells (Figures 1B and S1A). Cells were plated in 96-well plates, induced to differentiate by the addition of insulin, glucocorticoid, and cAMP stimuli, fixed at different time points after induction, and stained with antibodies to quantify the expression level of the key adipogenic transcription factors. When we averaged the resulting antibody intensities from cells fixed at each day of adipogenesis, we observed a sequential order of events, similar to previously published western blot results by Farmer (2006). In both OP9 cells and 3T3-L1 cells, maximal average C/EBPβ expression occurred 1–2 days after the induction of adipogenesis, preceding the induction of maximal PPARγ and C/EBPα expression, and followed after a delay by maximal lipid droplet formation (Figures 1C and S1B).

### Bimodal Induction of PPARγ, C/EBPα, and C/EBPβ

To further explore the relationship between the transcription factors, we carried out single-cell, multiparameter analysis (Figure S1C). In contrast to analysis of population averages, histograms that plotted the concentration of the transcription factors in each of approximately 25,000 single cells showed bimodal expression of PPARγ, C/EBPα, and C/EBPβ, starting at day 3 of adipogenesis even though the cells had been uniformly stimulated (Figure 1D). Just as separating proteins out on a two-dimensional gel results in better resolution than separating in a one-dimensional gel, using two parameters to plot the histograms better resolved the bimodal nature of the transcription factor induction. Each of the panels in Figure 1E shows a dual-parameter histogram analysis that plotted the frequency at each day of differentiation at which individual cells had a given concentration of PPARγ and C/EBPβ. At day 0, all the cells had low PPARγ and low C/EBPβ. At days 1 and 2, the cells had slightly higher PPARγ and significantly higher C/EBPβ than at day 0. At day 3, two populations of cells were clearly evident, indicating that sometime between days 2 and 3, a subpopulation of cells reverted back into a low PPARγ and low C/EBPβ state, whereas a second kept increasing their high PPARγ and high C/EBPβ level. Strikingly, this transition into the high PPARγ and high C/EBPβ state occurred early in adipogenesis, 1 day before accumulation of lipid, which is the usual marker of terminal differentiation.

Our population-averaged results shown in Figure 1C initially suggested that for adipogenesis to occur, C/EBPβ expression first drops at day 2 to about half before PPARγ and C/EBPα reach maximal expression (Yeh et al., 1995). However, single-cell analysis led to a different conclusion (Figure 1E, schematics). After removal of the glucocorticoid and cAMP stimuli, the expression of both C/EBPβ and PPARγ further increased in a fraction of cells, whereas in the remaining cells, the expression of both C/EBPβ and PPARγ dropped back to basal in both OP9 (Figure 1E) and 3T3-L1 cells (Figure S1D). Thus, even though all cells experienced the same differentiation-inducing stimulus, this remarkable switch behavior resulted in two groups of cells with distinct levels of PPARγ and C/EBPβ and with increasing amounts of differentiation inducers resulting in increasing numbers of cells in the high PPARγ-high C/EBPβ differentiated cell group (Figure S1E). This bifurcation suggested that each cell undergoes an all-or-none cell fate decision to either commit to differentiation or to revert to the low PPARγ-low C/EBPβ preadipocyte state.

### PPARγ Is Regulated by Two Positive Feedbacks to C/EBPα and to C/EBPβ

**Figure 2:**
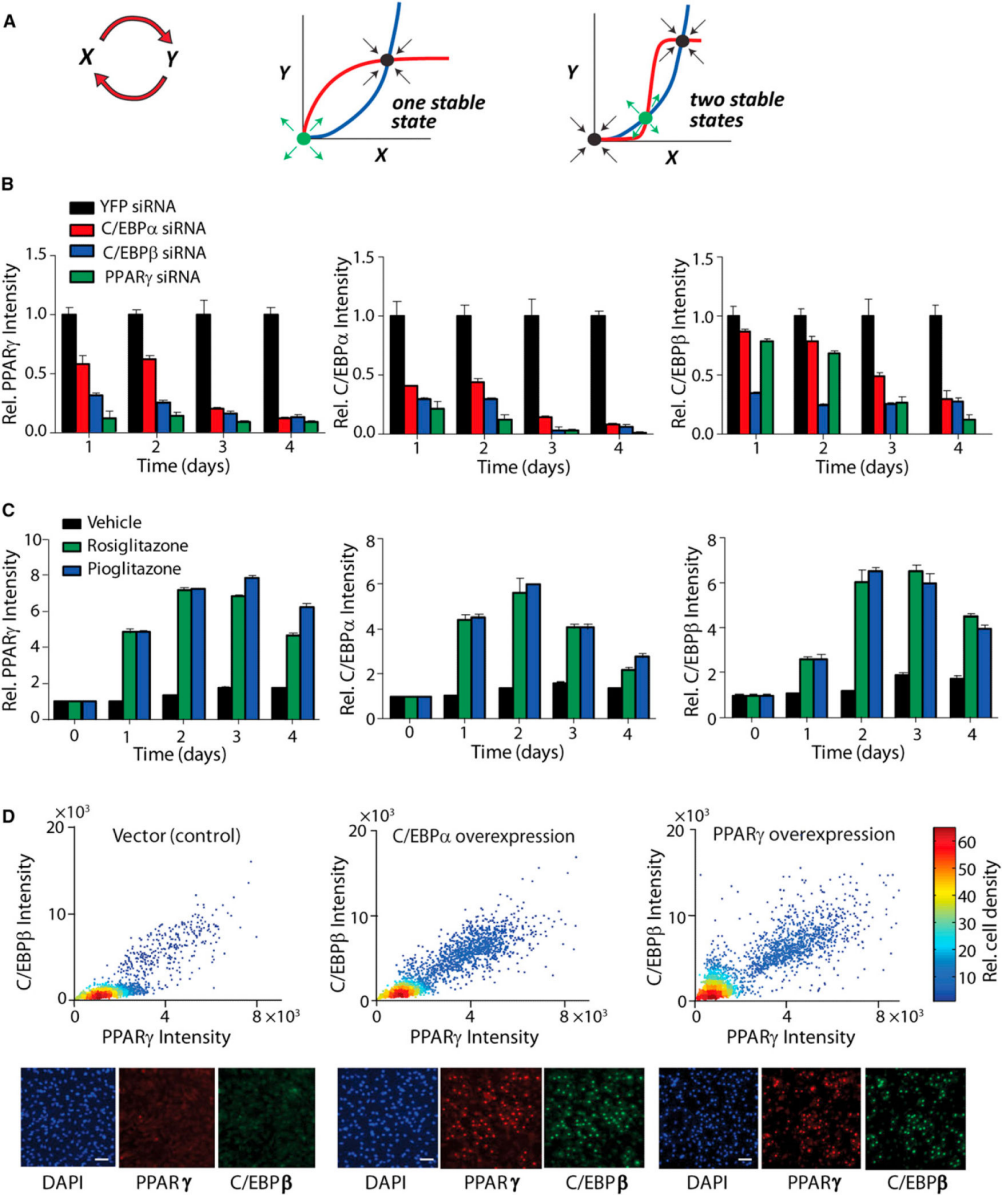
Identification of a Positive Feedback Loop between PPARγ and C/EBPβ (A) The left view is a schematic of a feedback loop between two variables x and y. The middle view is steady-state plots (dx/dt = 0 is in blue; dy/dt = 0 is in red) where the feedback loop from x to y and the feedback loop from y to x are both linear. When the feedback loops are both linear, there is only one stable steady state (black dot) and one unstable steady state (green dot). The right view is steady-state plots indicating where the feedback loop from x to y is still linear (blue), but now the feedback loop from y to x is highly cooperative (red). In this case there are two stable steady states and one unstable steady state. (B) OP9 cells were transfected with siRNA (20 nM) and 24 hr later were stimulated to differentiate with insulin, glucocorticoid, and cAMP stimuli. All values were normalized to the YFP (control) value at each time point. (C) Activating PPARγ with small molecules results in increased C/EBPα and C/EBPβ expression. Rosiglitazone (10 μM), pioglitazone (10 μM), or DMSO (control) was added to the media of undifferentiated OP9 cells. For (B) and (C), the cells were fixed at the respective time points, stained with antibodies to PPARγ, C/EBPα, and C/EBPβ, and analyzed by epifluorescence microscopy. Each bar represents approximately 20,000 cells from four separate wells (mean ± SD of four replicate wells). (D) Overexpression of C/EBPα or PPARγ by retroviral infection resulted in expression of C/EBPβ in the corresponding cells. Cells were fixed 10 days after transfection, costained with specific antibodies to PPARγ and C/EBPβ, and analyzed by epifluorescence microscopy. Scatterplots representing the correlation between C/EBPβ expression versus PPARγg expression are demonstrated. Lower panels show representative immunofluorescent staining of nuclei (blue), C/EBPβ (green), and PPARγ (red). Scale bars, 50 μm. See also Figure S2.

To enable the existence of two stable states, a system typically requires positive feedback, as well as one or more cooperative regulatory steps. Figure 2A shows a schematic representation of how positive feedback can help create such a decision. In the simplest case of positive feedback, the change in x is linearly related to the amount of y and vice versa. At steady-state conditions, the curves representing the dependence of x on y (blue line) and y on x (red line) will intersect at two points: one stable, and one unstable (Figure 2A, middle panel). If y is instead cooperatively related to x, for example if y is a gene that is only transcribed when three binding sites for transcription factor X in its promoter are occupied, this cooperative relationship can be described by a Hill equation and plotted as a sigmoidal curve (Figure 2A, right panel, red line). With this added cooperativity the curves representing the dependence of x on y (blue line) and y on x (red line) will now intersect at three points at steady-state conditions: two stable states and one unstable state. Positive feedback ensures that the system cannot rest in intermediate states, and cooperativity filters small signals out, allowing the system to have a stable off as well as a stable on-state (Ferrell and Xiong, 2001).

We first confirmed that a previously described positive feedback loop exists between PPARγ and C/EBPα (Rosen et al., 2002; Wu et al., 1999) by siRNA-mediated knockdown of PPARγ and C/EBPα expression, which reduced the expression of C/EBPα and PPARγ, respectively (Figure 2B, left and middle panels). However, one feedback loop—unless highly cooperative—is not enough to generate a bistable switch (Brandman and Meyer, 2008). We therefore searched for other potential feedback loops that could contribute to the bimodal patterns observed in Figure 1. As expected because C/EBPβ has been shown to act upstream of PPARγ (Yeh et al., 1995), knockdown of C/EBPβ reduced PPARγ expression (Figure 2B, left panel). However, when we used siRNA to suppress PPARγ expression, we found, especially at days 3 and 4, that C/EBPβ expression was also markedly reduced (Figure 2B, right panel), arguing that PPARγ was able to regulate the expression of its upstream activator C/EBPβ and suggesting that a second positive feedback links PPARγ and C/EBPβ. The existence of such a feedback loop is also supported by promoter binding studies that showed interactions of C/EBPβ with the PPARγ promoter (Schmidt et al., 2011) and of PPARγ with the C/EBPβ promoter (Mikkelsen et al., 2010).

Our siRNA data showed that this feedback loop between PPARγ and C/EBPβ engaged only 3–4 days after induction of adipogenesis, providing an important second boost to C/EBPβ expression. This suggested that a main role of this feedback is to keep C/EBPβ levels high after 48 hr independently of the initial glucocorticoid receptor (GR) and cAMP stimulation. This positive feedback between PPARγ and C/EBPβ therefore has the characteristics of a stabilizing switch mechanism that keeps PPARγ and C/EBPβ autonomously high even after the initiating stimulus is removed.

**Figure 3:**
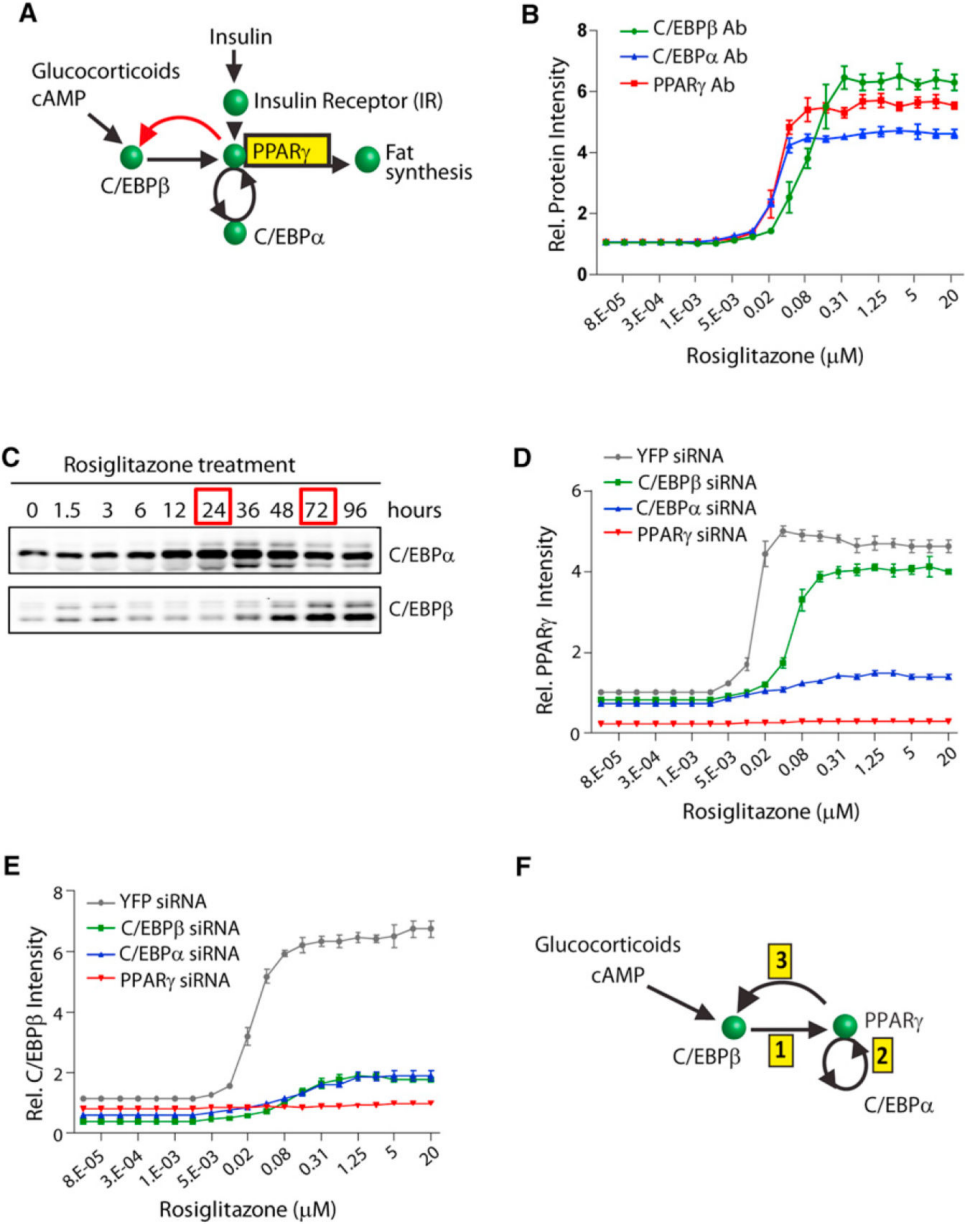
Characterization of the PPARγ-C/EBPβ and PPARγ-C/EBPα Feedback Loops (A) Diagram shows the here-identified feedback loop between PPARγ and C/EBPβ in red. (B) PPARγ, C/EBPα, and C/EBPβ expression in OP9 cells in response to increasing concentrations of rosiglitazone is illustrated. All values were normalized to basal values (without rosiglitazone). (C) One micromolar of rosiglitazone was added to the media of undifferentiated OP9 cells, and the cells were harvested at the indicated times. Then equal amounts of each protein sample were subjected to western blot analysis. (D and E) 20 nM of YFP (control), C/EBPβ, C/EBPα, or PPARγ siRNA was transfected into undifferentiated OP9 cells 24 hr prior to adding rosiglitazone. Cells were fixed 48 hr after adding rosiglitazone. All values are normalized to the value of YFP siRNA-transfected cells without rosiglitazone. For (B), (D), and (E), protein expression was quantified by immunohistochemistry staining of the cells with the respective specific antibodies and then imaging. Each data point represents ~20,000 cells (mean ± SD of three replicate wells). (F) Diagram shows the sequential order of steps that trigger the bistable switch. See also Figure S3.

To confirm the existence of a positive feedback from PPARγ to C/EBPβ by a second independent method, we used the PPARγ activators rosiglitazone and pioglitazone to directly activate endogenous PPARγ in the absence of other stimuli (Willson et al., 2001) (Figure 2C). Addition of both activators induced a marked upregulation of C/EBPβ (Figure 2C, right panel). Finally, we also overexpressed PPARγ using retroviruses to confirm that increasing PPARγ expression resulted in increased C/EBPβ expression. The images of the single-cell analysis, as well as a quantitative scatterplot of the single-cell results, show significant correlation between PPARγ and C/EBPβ expression (Figure 2D). We also confirmed the existence of the PPARγ-C/EBPβ feedback loop in the 3T3-L1 cell model (Figure S2D), which argued that this second feedback loop is a general mechanism for driving adipogenesis. Thus, as depicted in the scheme in Figure 3A, two consecutive positive feedback loops generate a bimodal distribution of high or low PPARγ, C/EBPβ, and C/EBPα activity early in adipogenesis.

### Consecutive and Cooperative Induction of the PPARγ-C/EBPα Followed by the PPARγ-C/EBPβ Positive Feedback Loops

The existence of feedback loops from PPARγ meant that we could trigger the expression of C/EBPβ, C/EBPα, and PPARγ just by adding a PPARγ activator, without needing glucocorticoids or increased cAMP. To determine whether the regulatory steps that initially induce the expression of PPARγ, C/EBPα, and C/EBPβ were cooperative, as would be predicted for a bistable system, we titrated the PPARγ activator rosiglitazone into the media of undifferentiated OP9 cells and monitored the resulting protein expression levels after 48 hr (Figure 3B), a time point at which all three transcription factors showed maximal expression (Figure 2C). Consistent with the existence of a cooperative step in the induction of C/EBPβ, C/EBPα, and PPARγ, the stimulus-response curves all had sigmoidal shapes that could be best fit with Hill coefficients of ~2.5.

Interestingly, the half-maximum response (EC_50_) for C/EBPβ expression was 4-fold higher than that for C/EBPα expression, indicating that the PPARγ-C/EBPβ feedback loop has a higher threshold for activation than the PPARγ-C/EBPα feedback loop. Because it takes time to build up the level of PPARγ, the positive feedback loop between PPARγ and C/EBPβ would be predicted to then engage with a delay after the PPARγ to C/EBPα loop. We confirmed that there is indeed a marked delay between engagement of the PPARγ-C/EBPα and PPARγ-C/EBPβ feedback loops. Western blot analysis performed at different time points after treatment with rosiglitazone showed that C/EBPα expression reached a maximal level within 24 hr (Figure 3C). However, maximal C/EBPβ expression was reached only after 72 hr.

Our analysis introduces a regulatory motif whereby a first feedback loop has to be engaged for a prolonged time period in order for a second feedback to be triggered that then carries the differentiation commitment process forward. A plausible result of such a second amplification by the PPARγ-C/EBPβ feedback loop is to create a sharper transition to the differentiated state. Indeed, when we used siRNA against C/EBPβ to suppress the second PPARγ-C/EBPβ feedback loop, the transition from low to high PPARγ expression with increasing amounts of rosiglitazone was more gradual and less robust compared to the sharp transition in the cells transfected with control YFP siRNA (Figure 3D). With the PPARγ-C/EBPβ feedback loop suppressed, a large population of the cells was unable to transition to the high PPARγ state, even when maximal doses of rosiglitazone were applied (Figure S3). Similar to other systems with multiple positive feedback loops (Brandman et al., 2005), the higher cooperativity generated by two consecutive feedback loops gives cells a stable off, as well as on, state (as described in Figure 2A). The stable off-state allows cells to reject short- or low-amplitude stimuli, which provides one of the key mechanistic ingredients for a sharp, all-or-none transition to a committed on-state.

To understand the respective roles of the different feedback loop components, we performed detailed siRNA-mediated perturbation experiments. Knockdown of C/EBPα resulted in an almost-complete suppression of PPARγ expression (Figure 3D), confirming that the PPARγ-C/EBPα feedback loop was essential for PPARγ expression (Rosen et al., 2002). Knockdown of C/EBPα also resulted in almost-complete knockdown of C/EBPβ expression (Figure 3E), showing that the PPARγ-C/EBPβ feedback loop required the presence of a functioning PPARγ-C/EBPα feedback loop and arguing for the sequential induction model depicted in Figure 3F: (1) cAMP and glucocorticoid signals initially drive C/EBPβ expression; (2) increasing C/EBPβ above a critical threshold then triggers the start of a positive feedback between PPARγ and C/EBPα that, after a time delay; (3) induces the second PPARγ-C/EBPβ feedback loop so that most of the cells transition into a terminally differentiated state. However, there was always a fraction of cells falling back to the basal state (Figure 1E), raising the question how cells regulate which fraction becomes locked in the differentiated state.

### Characterization of a Late-Acting, Third Positive Feedback Loop between PPARγ and the Insulin Pathway

**Figure 4:**
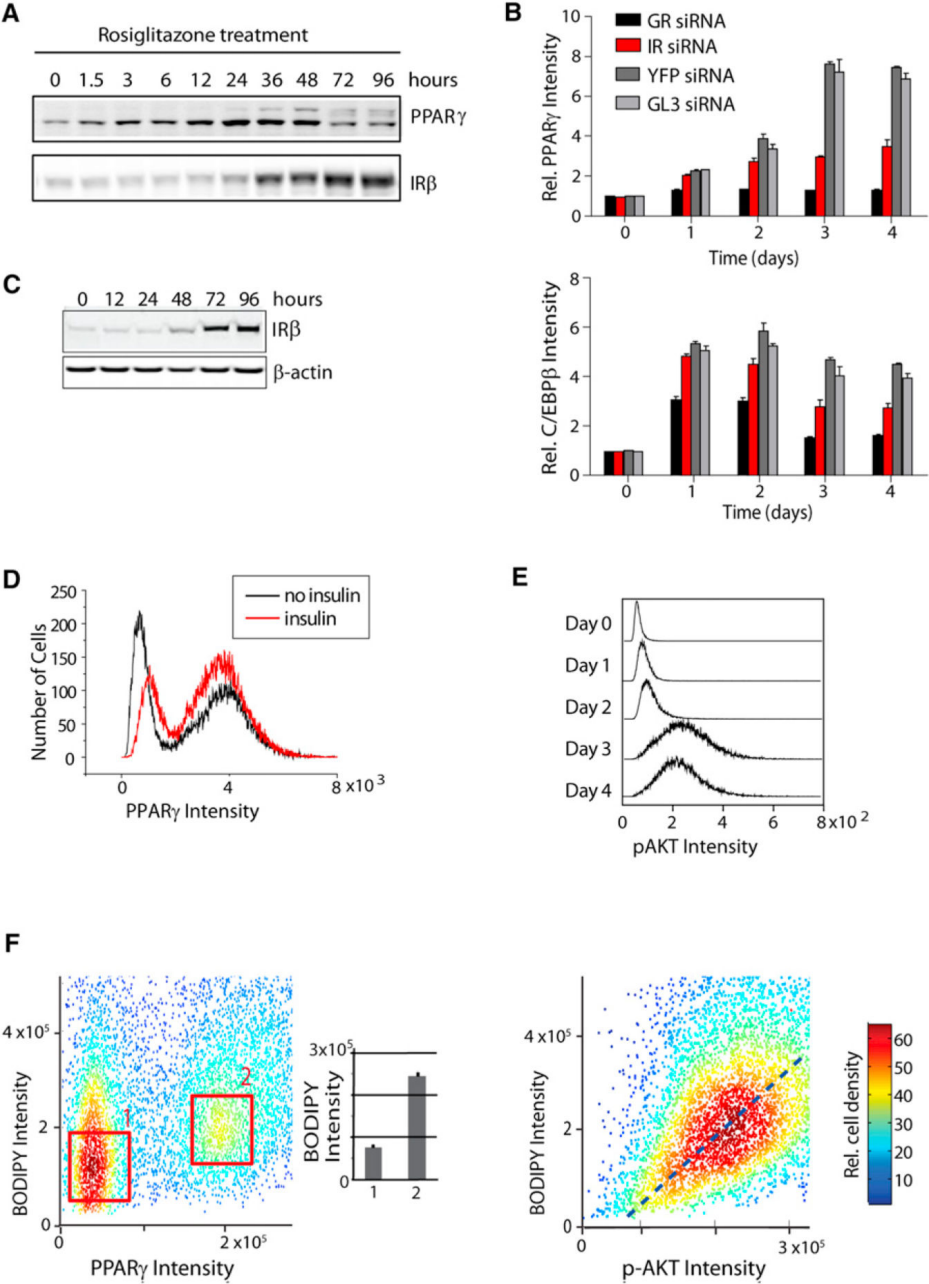
Characterization of a Third, Late-Acting Feedback Loop between PPARγ and the Insulin Pathway (A) Time course of PPARγ and insulin receptor (IR) expression in OP9 cells in response to rosiglita-zone addition is presented. (B) 20 nM of glucocorticoid receptor (GR), IR, or control (YFP, GL3) siRNA was transfected into undifferentiated OP9 cells that were, 24 hr later, stimulated to differentiate. C/EBPβ and PPARγ expression levels were measured by single-cell immunohistochemistry using specific antibodies. Each bar represents 7,000 single cells (mean ± SD of four replicate wells). All values were normalized to the value of the YFP siRNA-transfected cells at day 0. (C) Western blot shows IRβ expression over the time course of adipogenesis. (D) Histograms show number of cells (y axis) with the specified concentrations of PPARγ (x axis) with 175 nM insulin or without insulin at day 3. (E) Histograms show number of cells (y axis) with the specified concentrations of pAKT (x axis). Approximately 25,000 cells were stained with pAKT(S473) antibody and analyzed for each histogram. (F) Scatterplot shows concentrations of BODIPY versus PPARγ or p-AKT in ~7,000 individual OP9 cells 96 hr after the induction of adipogenesis. As shown in the inset bar plot, cells at the center of the high PPARγ population (box labeled ‘‘2’’) had an ~3× higher average BODIPY intensity than cells at the center of the low PPARγ population (box labeled “1”). For (B–F), undifferentiated OP9 cells were induced to differentiate by adding the adipogenic cocktail for 2 days and then replacing the medium with fresh growth medium containing 175 nM insulin and 10% FBS. See also Figure S4.

We observed that upregulating PPARγ activity increased insulin receptor expression (Figure 4A), which was not surprising since an earlier study had shown that C/EBPα can regulate insulin receptor expression and PPARγ and C/EBPα are in a positive feedback loop (Wu et al., 1999). To test whether the converse were true—that the insulin pathway could regulate PPARγ expression, thus creating a third feedback loop from PPARγ via C/EBPα—we used siRNA to knock down insulin receptor expression and carried out the standard adipocyte differentiation protocol. Indeed, insulin signaling is required to increase PPARγ expression 2-fold between days 2 and 3 (Figure 4B). However, during the first 2 days of adipogenesis, insulin signaling has only a small effect on C/EBPβ and PPARγ expression (Figures 4B and S4A), most likely due to the fact that the insulin receptor is strongly expressed only after day 2 (Figure 4C). As a control in the siRNA experiments, knockdown of the GR, which is needed to start differentiation, suppressed C/EBPβ and PPARγ expression already at day 1 (Figure 4B). These results confirm the existence of a third feedback loop between PPARγ and the insulin receptor that only engages with a delay after the induction of the C/EBPβ-PPARγ-C/EBPα dual-positive feedback system (Figures 3C and 4A).

This third feedback loop between PPARγ and the insulin pathway is not needed to trigger the bistable switch as evidenced by the fact that the switch triggers regardless if insulin is added under normal differentiation induction with glucocorticoids and cAMP (Figure 4D) and that rosiglitazone stimulation induces the switch even though no insulin is added (Figure S3). Furthermore, pAKT levels that can be used to monitor insulin signaling are never bimodal during adipogenesis (Figure 4E), in contrast to the bimodal expression of the switch components C/EBPβ, C/EBPα, and PPARγ (Figure 1D). As shown in Figure 4D, the main function of this third feedback loop between PPARγ and the insulin receptor is to amplify and boost PPARγ expression after the switch is made. Thus, the consecutive action of the three positive feedback loops generates a subpopulation of differentiated cells with persistently high PPARγ levels.

### Insulin Signaling Controls Fat Accumulation in Differentiated Cells in a Graded Fashion

To directly determine the relationship between PPARγ expression and lipid accumulation, we used immunohistochemistry to measure both parameters in the same individual cells. Because insulin signaling is a main regulator of adipocyte metabolism, we further monitored the insulin receptor pathway by measuring the cellular intensity of p-AKT. The inset plot in Figure 4F (left) shows that the differentiated population with high PPARγ had on average ~3-fold more lipid incorporated than the low PPARγ population, consistent with the interpretation that a persistently high level of PPARγ defines the differentiated adipocyte state. However, at the single-cell level, fat accumulation correlated only weakly with relative PPARγ expression (Figure 4F, left). This can be seen by the wide spread of BODIPY intensities when focusing on cells with high PPARγ. Thus, whereas PPARγ drives the differentiation process and marks differentiated cells, fat accumulation itself must be under control of another signaling pathway. Strikingly, the degree of lipogenesis in these differentiated cells with high PPARγ could be more closely predicted by the relative activity of p-AKT (Figure 4F, right), arguing that the strength of insulin-Akt signaling is the main determinant of how much fat is accumulated in an individual differentiated cell. The same data further show that this relationship between p-AKT and fat accumulation is graded, not bistable, which means that once the switch is made into the differentiated, high PPARγ state and high levels of insulin receptors are present, there is no threshold that needs to be overcome to accumulate fat. Weak insulin stimuli will already cause some fat formation, and increasing the insulin stimulus will proportionally increase the amount of fat synthesis in existing adipocytes. Of note, there was a small fraction of the total cell population that had high BODIPY intensity (Figure 4F, left), and also high p-Akt, whereas having low PPARγ expression. These cells may have alternative regulatory mechanisms to increase insulin signaling not involving PPARγ expression.

### Development of a Quantitative Molecular Model of Adipogenesis

**Figure 5:**
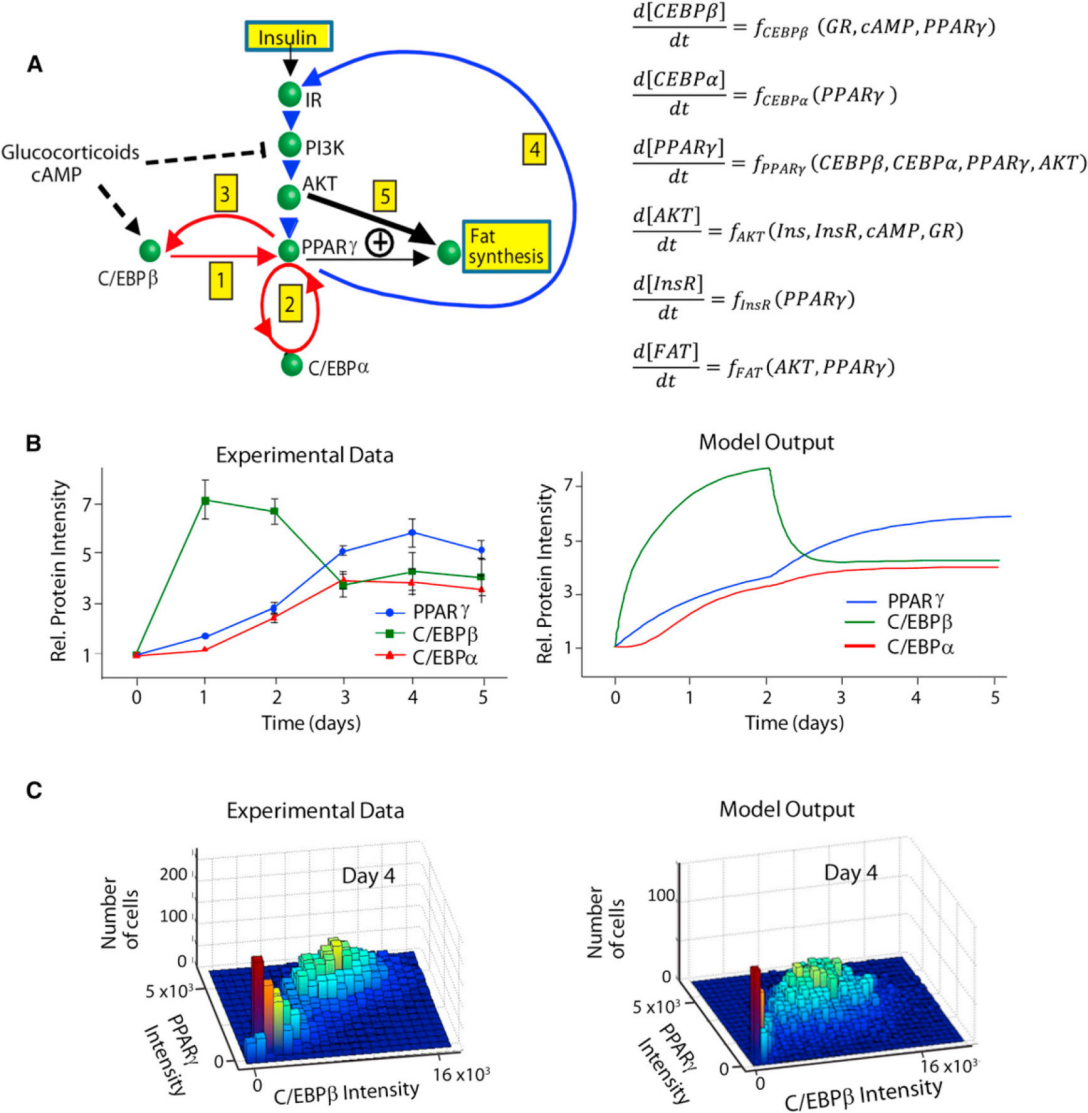
Development of a First Quantitative Molecular Model of Adipogenesis (A) The left view is a diagram depicting the sequence of steps leading to a terminally differentiated fat cell and subsequent accumulation of lipid. The dashed lines show the activating and inhibiting roles of cAMP and glucocorticoids (see also Figures S4B–S4E). The heavy, double-lined black arrows indicate that lipogenesis is much more strongly correlated with p-AKT activity than with PPARγ expression. The model equations are shown on the right. (B) Output of the model compared to the experimental data from Figure 1B is presented. (C) Stochastic variation in the rates of C/EBPβ, C/EBPα, and PPARγ expression levels causes two subpopulations of cells to exist even for a uniform stimulation. See also Figure S5.

We used the data from our single-cell analysis to generate a quantitative model of the C/EBPβ-PPARγ-C/EBPa-driven bistable switch, the insulin receptor-mediated PPARγ booster mechanism, and the subsequent insulin-regulated lipogenesis program. The diagram in Figure 5A illustrates the consecutive order of the three positive feedback loops that we identified for PPARγ activation (marked as steps 2, 3, and 4, respectively) and the subsequent insulin control of lipid accumulation (marked as step 5). Of note, there is likely no direct positive feedback between C/EBPα and C/EBPβ because chromatin immunoprecipitation data sets (Schmidt et al., 2011; Siersbæk et al., 2011) showed no evidence of C/EBPα or C/EBPβ binding to each other's promoters. In addition we also included in our model the previously demonstrated inhibition of the insulin signaling pathway by glucocorticoid and cAMP (Li et al., 2008), which we confirmed in experiments shown in Figures S4B–S4E. From a conceptual perspective the adipocyte differentiation system represents a novel design with consecutive positive feedbacks that engage at different times during the commitment process, allowing cells to ultimately reach a terminally differentiated state. The model recreates the initial increase in C/EBPβ expression, followed by the upregulation of C/EBPα and PPARγ, that is seen when thousands of single-cell measurements are averaged (Figure 5B, left; reproduced from Figure 1C).

To take into account that expression levels of regulatory proteins vary between individual mammalian cells (Niepel et al., 2009), we added stochastic variations to the relative amplitude of PPARγ, C/EBPβ, and C/EBPα synthesis, degradation, and basal expression parameters, respectively. As shown in Figure 5C when an average of 30% lognormal noise was added to the parameters, the model replicated the bimodality observed experimentally in Figure 1E, providing in silico evidence that the adipogenesis system is inherently bistable. No matter how we varied the initial parameters and pulled away from median values, the system always reverted back to one of the two stable points schematically shown in Figure 2A (right panel), which represent a stable differentiated and a stable nondifferentiated state.

This same analysis can also be used to estimate differences in the intrinsic noise among the PPARγ, C/EBPα, and C/EBPβ parameters. A best match was observed when PPARγ rates were varied less than the C/EBPβ and C/EBPα rates—by an average of 15% for PPARγ compared to 30% for C/EBPβ and C/EBPα, respectively (Figure S5A). The modeling further shows that if the protein variation would be much smaller, e.g., 3%, there would not be sufficient variation to create two populations of cells (Figure S5B). All cells would either remain undifferentiated, or all would switch to the differentiated state as the stimulus increases. On the other hand, if the variation were 100%, most cells would be in a state where the bistability of the system would break. The sweet spot in variation where bimodality is generated without breaking the system is approximately 15%–45%.

Thus, both experiments and modeling demonstrate that a uniform stimulus can create distinct differentiated and nondifferentiated subpopulations of cells with high versus low PPARγ/C/EBPβ concentrations, respectively. This induction of two clearly separate subpopulations can be explained by stochastic variation of the expression levels of the key regulatory proteins. Whether or not a particular “cell” will fall into the low or high PPARγ and C/EBPβ subpopulation depends on whether the relative expression levels of the regulatory proteins position the cell below or above a system's threshold where the bistable switch is triggered. Because they are connected by feedback, all three regulatory proteins contribute to setting the threshold of the system. However, consistent with a more central role of PPARγ in controlling the threshold, model calculations showed that expression of PPARγ immediately before the switch is triggered is more predictive of a cell's subsequent differentiation state compared to the levels of C/EBPβ or C/EBPα (Figure S5C).

### Multiple Consecutive Positive Feedbacks Are Required to Create an Irreversible, Committed Differentiation State

**Figure 6:**
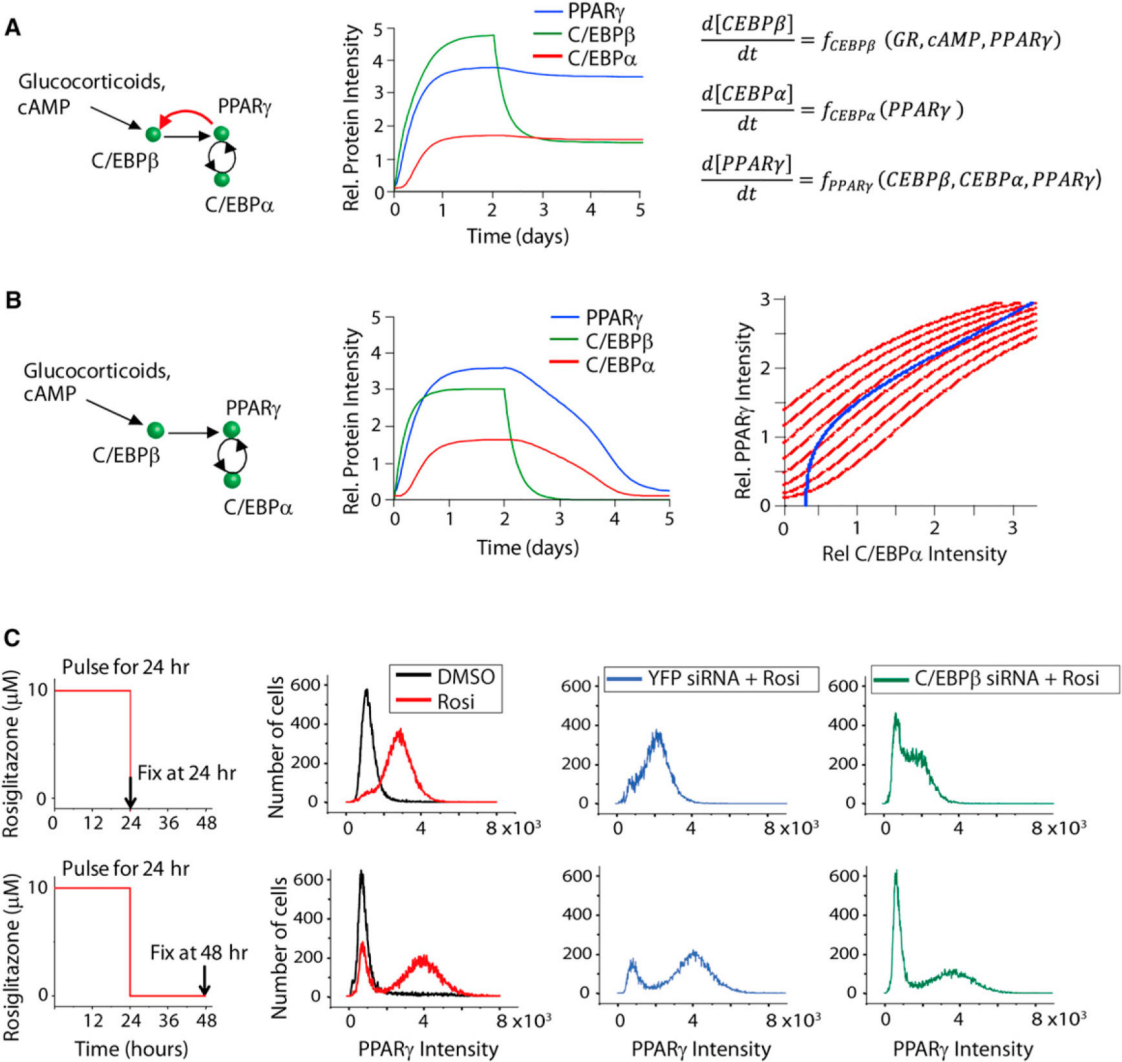
Consecutive Positive Feedback Is Required to Create an Irreversible, Committed Differentiation State (A) Schematic, model output, and model equations for the two-feedback loop bistable switch are presented. (B) Schematic, model output, and steady-state plot for a one-feedback loop system are presented. To generate the steady-state curves, the equations for d[PPARγ]/dt and d[C/EBPα]/dt in the model were set to zero and plotted. Incrementally increasing values of constant C/EBPβ were used to generate each of the PPARγ steady-state curves (red). The C/EBPα steady-state curve is shown in blue. (C) Experiment to test whether a one-feedback loop system can create a bistable transition. Each histogram represents PPARγ nuclear intensities from approximately 30,000 cells. At time 0, undifferentiated OP9 cells were stimulated with rosiglitazone (Rosi; 10 μM) for 24 hr or left in basal media, then washed three times with fresh medium, and then either fixed or placed in fresh medium without rosiglitazone for 24 hr and then fixed. For the siRNA experiments, C/EBPβ or YFP siRNA was introduced into OP9 cells by reverse transfection 24 hr before time 0.

We next tested whether the model reproduces our earlier observation in Figures 4B and 4D that the initial glucocorticoid and cAMP stimulation is sufficient to lock the system into a committed state even without the third positive feedback to the insulin receptor. The output of these simulations shows that PPARγ, C/EBPα, and C/EBPβ stayed high even after the glucocorticoid and cAMP stimuli were removed after 48 hr, demonstrating that the switch can be triggered even without the third positive feedback loop between PPARγ and the insulin receptor (Figure 6A). However, if the second feedback loop between PPARγ and C/EBPβ was removed from the model, PPARγ, C/EBPα, and C/EBPβ levels fell back down to their initial low values after glucocorticoid and cAMP stimuli were removed (Figure 6B). Plotting the steady-state curves (Figure 6B, right) showed that in a system with just one feedback loop, there is not much cooperativity, and thus, the steady-state curves do not have much sigmoidal bending. As a result, there are very few points in the parameter space where the steady-state curves can intersect and where the system can maintain two stable states.

To experimentally test whether or not a one-feedback loop model can trigger an irreversible transition, we used siRNA to knock down the expression of C/EBPβ versus control (YFP siRNA) and then added rosiglitazone to activate the feedback loops. As shown in Figure 6C, stimulating with rosiglitazone for 24 hr partially increased PPARγ. Then when the stimulus was removed for 24 hr, about half the cells locked into the differentiated, high PPARγ state, and the other cells fell back into the undifferentiated state. However, if the PPARγ-C/EBPβ is suppressed by siRNA knockdown, many cells could not sufficiently increase PPARγ, and even if they did, the majority fell back into the undifferentiated state when the stimulus was removed for 24 hr. As we show computationally and experimentally, the failure to maintain a committed state in a one-feedback loop system demonstrates why the increased cooperativity provided by the second positive feedback between PPARγ and C/EBPβ is of utmost importance in creating a robust bistable system.

### History Dependence or Hysteresis of the Positive Feedback Loops

An important additional characteristic of a predicted bistable system is hysteresis. Hysteresis can be demonstrated by using an initial strong stimulus to lift cells into an on-state and then returning the cells to a low stimulus level that had previously kept the cells in the off-state. If a system has hysteresis, even though the stimulated cells have returned to a low stimulus level, they do not turn off. Rather, cells remain stuck in the on-state. A system with hysteresis thus has biochemical memory, and cells are capable of “remembering” that they have been stimulated even though the stimulus has been withdrawn. We observed this important hysteresis characteristic in our computational adipocyte differentiation model when we applied a transient pulse of glucocorticoid and cAMP (Figure 6A). Hysteresis was evident by the sustained elevation of PPARγ even after the stimulus was removed.

**Figure 7:**
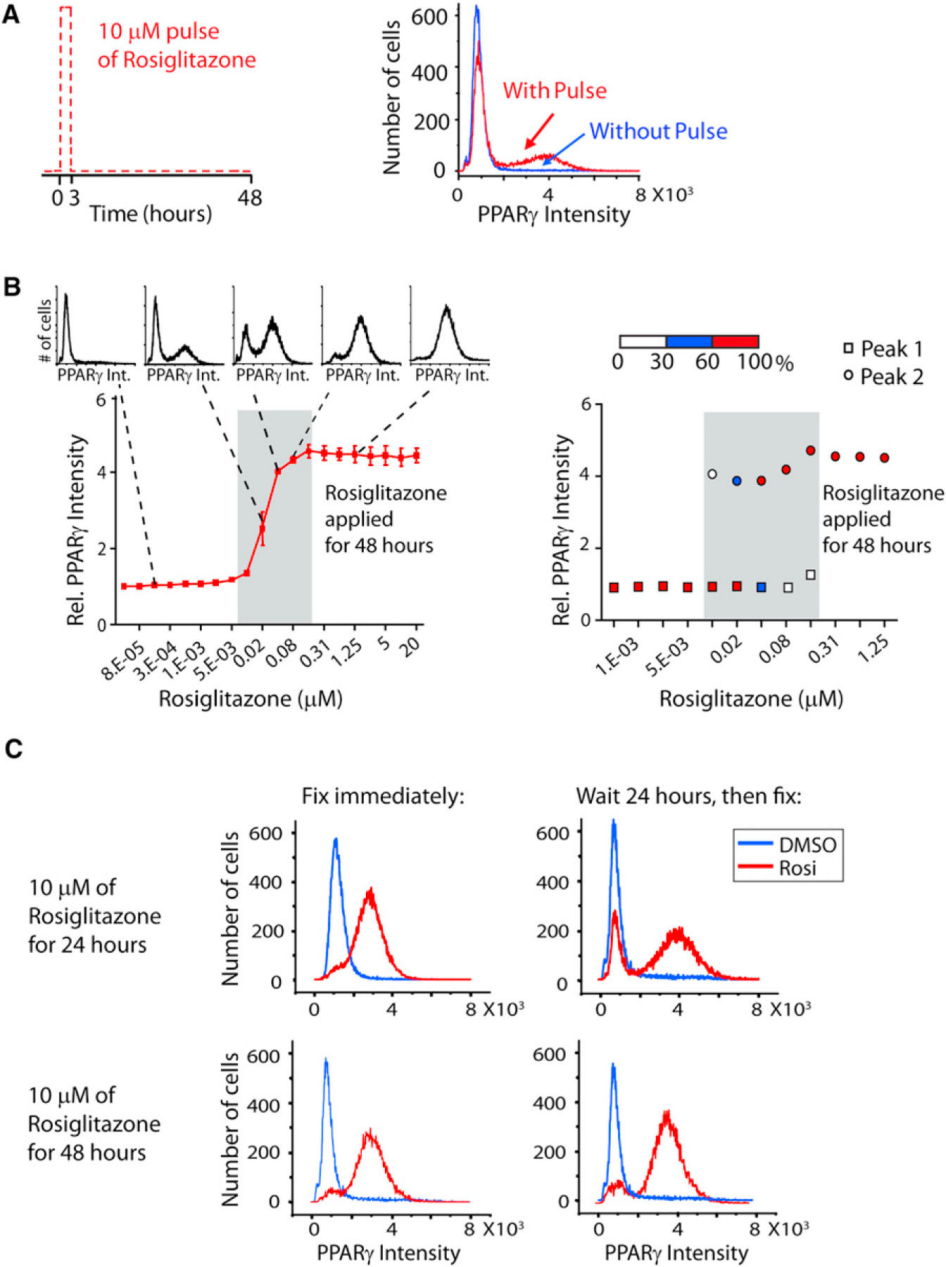
Using a Small Molecular Activator of PPARγ to Demonstrate Hysteresis in the Circuit Controlling Adipogenesis (A) A short, high-amplitude pulse of PPARγ activation can lock a fraction of cells in the differentiated state. At time 0, undifferentiated OP9 cells were either stimulated with rosiglitazone (10 μM) or control (DMSO) for 3 hr, washed three times with fresh medium, and then placed in fresh medium without rosiglitazone or DMSO. Cells were fixed 48 hr after treatment with a rosiglitazone pulse (red curve) or without a pulse (blue curve). Each histogram plots the nuclear PPARγ intensities from approximately 20,000 cells. (B) Increasing the amplitude of the PPARγ activation pulse locks more cells in the differentiated state. PPARγ expression versus rosiglitazone concentration is shown as a plot where each data point is the average of approximately 20,000 cells (±SD of triplicate wells, left) or as the change in distribution between the low PPARγ peak or the high PPARγ peak (right). Two-fold serial dilutions of rosiglitazone were added to the media of undifferentiated OP9 cells, and the cells were fixed 48 hr later. Protein expression was quantified by immunohistochemistry staining of the cells with the respective specific antibodies and then imaging. The horizontal white, blue, and red bar in the right plot shows the percentage of cells in the low or the high PPARγ peak for a given concentration of rosiglitazone. (C) A requirement for sustained PPARγ helps to prevent accidental triggering of the bistable switch. Even after 24 hr of rosiglitazone treatment, a large fraction of cells can still drop back to the low PPARγ, undifferentiated state when the stimulus is removed (top plots). Most cells only lock into the high PPARγ, differentiated state after 48 hr of sustained PPARγ activity (bottom plots). See also Figure S6.

Figure 7A shows an experimental test for hysteresis in response to direct activation of PPARγ. A brief 3-hr-long pulse of PPARγ activity induced by adding rosiglitazone to the media, followed by a return to nonstimulated conditions, was sufficient to keep a subset of the cells in the high PPARγ state even after the stimulus was removed for almost 48 hr. The blue trace in Figure 7A is shown as a control where the cells were not subjected to the pulse of PPARγ activity, and no cell converted to the differentiated state even though the PPARγ activity was the same from 3 to 48 hr as for the rosiglitazone-pulsed cells. Figure 7B shows that the adipogenesis differentiation system is indeed capable of hysteresis, meaning that there is a discontin uous jump from the low-to-high PPARγ state. When all the cells in a well are averaged together, one observes a continuous sigmoidal curve (Figure 7B, left panel). However, as shown by the five inset plots in Figure 7B, each point in this curve is actually the average of a population of cells that is either in the low PPARγ state or high PPARγ state. If instead of averaging all the cells, one plots the percentage of cells in each population as a function of PPARγ activity, the discontinuity in the stimulus-response relationship becomes apparent with cells being in one of two possible PPARγ intensity states for a given intermediate stimulus (grey-shaded area in Figure 7B). However, when maximal rosiglitazone is applied for 48 hr, all cells switch into the high PPARγ state.

Consistent with the existence of hysteresis, increasing the stimulus duration locks more cells into the high PPARγ state (Figure 7C). When C/EBPα, C/EBPβ, and PPARγ levels were monitored in response to PPARγ activity pulses ranging from 3 to 48 hr (Figure S6), the fraction of cells that end up in the differentiated state gradually increases. Together with the amplitude dependence in Figure 7B, these results argue that both, the amplitude as well as the duration of the activation pulse, jointly control the probability of a cell transitioning to the high PPARγ differentiation state.

Finally, the observed hysteresis in Figure 7 demonstrates that the rosiglitazone-induced transition to the high PPARγ state transition does not bypass the bistable switch mechanism described in Figure 1E but rather induces the same circuit. Thus, the same bistable consecutive positive feedback circuit with hysteresis is induced by stimulation either with glucocorticoid and cAMP or by direct activation of PPARγ, further arguing that this triple feedback circuit is the core mechanism that converts preadipocytes to terminally differentiated adipocytes.

## DISCUSSION

### Three Consecutive Positive Feedbacks Drive Preadipocyte-to-Adipocyte Differentiation

Our results demonstrate that a commitment decision is made by preadipocytes early in adipogenesis before the appearance of lipid droplets, which has been a previous criterion for defining a terminally differentiated adipocyte state. This commitment process is bistable rather than graded. We demonstrated that the commitment decision relies on three consecutive positive feedback loops: a first loop between C/EBPα and PPARγ, followed by a second loop between PPARγ and C/EBPβ, and a third positive feedback between PPARγ and the insulin receptor. Importantly, we found that the second feedback loop back to C/EBPβ only engages at a higher PPARγ level. The requirement for higher PPARγ caused a marked delay in the activation of the PPARγ-C/EBPβ loop compared to the PPARγ-C/EBPα loop. A third positive feedback loop between PPARγ and the insulin receptor then further boosts PPARγ expression and helps to maintain and consolidate the terminally differentiated state. This third positive feedback only engages after an additional delay forced by the need for insulin receptors to be expressed at a higher level and by cAMP and glucocorticoid suppression of the insulin signaling pathway, which is only removed late in the differentiation process (Figure S4). Together, the successive triggering of three positive feedbacks forces a sequence of predefined events onto the adipocyte differentiation process.

We demonstrated that the same differentiation switch can be induced by either the glucocorticoid and cAMP-mediated induction of C/EBPβ or, more directly, by the rosiglitazone-mediated activation of endogenous PPARγ. Both stimuli show hysteresis, have the same bimodality in the induction of the high C/EBPβ and PPARγ state, and have the same consecutive order of activation (Figure 7). The identical consecutive activation by the two different stimuli argues that the same feedback circuit design is responsible for the endogenous, as well as drug-induced, differentiation of adipocytes. This has mechanistic implications, suggesting that the same consecutive positive feedback loop circuit design can be triggered by different physiological or drug-induced stimuli, arguing that the circuit we identified is the core module responsible for fat cell differentiation.

The rosiglitazone experiments in Figure 7 demonstrated that the PPARγ-C/EBPα-C/EBPβ bistable switch can sense and transduce both the duration, as well as the amplitude, of the activating pulse into differentiating an increasing fraction of the cells while rejecting weak stimuli. This bistable switch provides a stable off-state that allows preadipocytes to exist for long periods of time in an undifferentiated state as long as the stimuli that activate PPARγ stay below a critical threshold and helps to explain how only a small fraction of adipocytes are renewed in an adult human every year (Spalding et al., 2008). In contrast, when the inducing stimuli are above the threshold, the fraction of cells converted to adipocytes can be controlled in a graded fashion (over about a factor of 16 in rosiglitazone in Figure 7B), allowing for better control of the number of adipocytes than would be obtained in a system in which all preadipocytes convert to adipocytes in an all-or-none fashion once a single critical threshold is crossed.

How can one explain why only a part of the cell population converts to the differentiated state when stimuli have submaximal amplitude and/or duration? If one assumes that the cells are identical and that differentiation is an all-or-none process at the level of single cells, all cells should differentiate for stimuli above a particular threshold value or all cells should remain undifferentiated for stimuli below that threshold value. As shown in Figure 7B, there is a range of low-amplitude stimuli where no differentiation is observed, followed by a range of intermediate-amplitude stimuli (shaded in grey) where the fraction of cells that differentiates increases in a graded fashion, followed by a range of high-amplitude stimuli where all cells are converted to the high PPARγ differentiated state. How can we reconcile an all-or-none differentiation switch with the observed graded response in the grey box? As demonstrated by model calculations for differentiation induced by glucocorticoid and cAMP stimuli (Figure 5C), the partial conversion of a cell population can be explained by cell-to-cell variability in expression levels of regulatory components, which results in variable sensitivity to PPARγ within an otherwise homogenous population of cells. Thus, for a given submaximal stimulus concentration, some cells will convert to the high PPARγ state sooner than others. The same fractional conversion also applies to increases in the duration of maximal stimuli (Figure S6).

Together, these results argue that organisms employ a system that combines consecutive positive feedback and stochastic variation and then use both the amplitude and the duration of the activating stimulus to control the number of differentiated adipocytes. The demonstrated requirement for persistent and strong inductive signals confers robustness to the system by preventing short- and low-amplitude stimuli from accidentally triggering differentiation.

### Graded Control of Fat Accumulation in Individual Differentiated Adipocytes by Insulin

A consequence of this irreversible commitment step early in differentiation is that cells must exist that are already committed to becoming fat cells but do not yet have a visible increase in fat storage. Once they switch into the persistently high PPARγ state, these differentiated adipocytes control the degree of lipogenesis in a graded fashion with fat accumulation closely correlating with the strength of insulin pathway activity in each cell (p-AKT; Figure 4). In contrast the relative level of PPARγ, which can vary in the differentiated cells, only weakly correlates with fat accumulation. Thus, two important regulatory programs need to be distinguished in the management of the weight of mammals: the regulation of the degree of fat storage in individual adipocytes, and the total number of adipocytes per organism. This total number of adipocytes changes only slowly with about 10% turnover per year in humans (Spalding et al., 2008). Our study then argues that a primary role for the insulin pathway is to directly control how much fat is stored in individual cells rather than regulating the differentiation decision. In contrast, the differentiation decision, which controls the number of fat cells, is under a mixed control of glucocorticoid and cAMP stimuli with only a minor contribution from insulin signaling.

### A Computational Molecular Model for Adipocyte Differentiation

In order to mechanistically understand the differentiation regulatory circuit, we developed a computational model for the conversion of preadipocytes to adipocytes. Specifically, our goals in modeling were to (1)learnmore about whycells use a consecutive multipositive feedback circuit design to control differentiation, and (2) understand our experimental observation of bimodality and how submaximal stimuli could convert only a fraction of stimulated preadipocytes to adipocytes. As a third, more long-term goal, we were interested to use differentiation models to predict which regulatory inputs are best suited as drug targets to regulate the total number of human adipocytes.

We showed that a single positive feedback loop with low cooperativity can amplify signals but cannot generate the observed robust bistability. The experimentally identified consecutive feedbacks make the differentiation process more nonlinear (ultrasensitive) and the switch more robust. Our model explains and quantitatively recapitulates how these sequential feedback loops are engaged to drive the preadipocytes into a persistent state characterized by elevated PPARγ, C/EBPα, C/EBPβ, and insulin receptor expression. This example of a differentiation process provides a conceptual framework that a sequential positive feedback circuit design is well suited to induce a robust transition to a differentiated state. It is suggestive to propose that similar consecutive positive feedback circuit designs drive many, if not most, other differentiation processes.

Our model further demonstrated how stochastic variations in the expression levels of C/EBPβ, C/EBPα, and PPARγ cause a differentiating and nondifferentiating population to coexist even though all cells are subjected to the same stimuli. This finding from the model can explain in molecular terms why intermediate stimuli only convert a fraction of preadipocytes into differentiated cells rather than generating potentially undesirable and misfunctioning partially differentiated cells. This single-cell variation concept also provides a molecular explanation of how organisms can have robust, all-or-none conversion of individual preadipocyte cells into adipocytes, whereas at the same time ensuring that the total population of preadipocytes does not convert in an all-or-none fashion for intermediate stimuli. Rather, single-cell variation ensures that only a small fraction of the total preadipocyte population converts everyday to an irreversible, differentiated adipocyte state (Spalding et al., 2008).

Finally, understanding if, when, and where a clear commitment decision is made by preadipocytes is critical for knowing how to target therapeutics to manipulate the differentiation process as a possible means to treat obesity, diabetes, and other adipocyte-associated diseases. Because the signaling and transcriptional network controlling adipogenesis involves multiple feedbacks with different time constants, it cannot be readily understood by a graphical diagram alone. We argue that our quantitative molecular working model of adipogenesis can be used to guide experiments, to conceptually understand the induction process, and also to have a new way to test the effect of different inputs and perturbations to components in the network. As an example of such a use of the model to predict outcomes, our experiments in Figure 7 confirmed the prediction from the model that direct activation of PPARγ by rosiglitazone should induce the bistable switch with coinduced C/EBPα, PPARγ, and C/EBPβ expression rather than bypassing the switch and directly regulating the adipogenesis-relevant genes downstream of PPARγ. The latter could have been predicted with equal plausibility. Instead, our study demonstrated that direct PPARγ activation by rosiglitazone triggers the same consecutive positive feedback circuit that is triggered by glucocorticoid and cAMP signaling. The ability of the model to predict drug action exemplifies a future use of such differentiation models to predict optimal combinations of potential therapeutic interventions to control the total number of adipocytes in a patient by increasing or decreasing the rate of differentiation from preadipocytes to adipocytes.

### Conclusions

Because of low cooperativity in typical single transcriptional feedback loops, multiple feedback loops are required in adipogenesis to generate sufficient cooperativity to reliably convert to the differentiated state. Importantly, the circuit design identified here with consecutive positive feedback loops, including one that reaches back to C/EBPβ, ensures that the differentiation decision is not triggered accidentally by uncoordinated or brief and low-amplitude hormonal stimuli. Our model and experimental analysis further show how stochastic variation in the expression of regulatory proteins is sufficient to explain how a submaximal stimulus triggers an all-or-none terminal differentiation of only a fraction of proliferating precursor cells. Together, our study provides conceptual insights into the adipogenesis process that likely applies to many, if not most, cell fate decisions.

## EXPERIMENTAL PROCEDURES

### Cell Culture, Differentiation, Transfection, Antibodies, and Plasmids

OP9 and 3T3-L1 cells were cultured according to the protocols in Wolins et al. (2006). OP9 cells were grown in growth medium consisting of MEM-α, 2 mM l-glutamine, 100 U/ml penicillin, and 100 μg/ml streptomycin, plus 20% FBS. 3T3-L1 cells were grown in 3T3-L1 propagation medium: DMEM with 10% bovine calf serum, 2 mM l-glutamine, 100 U/ml penicillin, and 100 μg/ml streptomycin. To induce differentiation, confluent cells were treated with a differentiation medium containing growth medium plus the standard adipogenic cocktail (DIM): 1 μM dex, 175 nM insulin, 0.5 mM IBMX, and 10% FBS. After 48 hr, the differentiation medium was replaced with growth medium, plus 175 nM insulin and 10% FBS. Diced pool siRNA was generated as previously described by Galvez et al. (2007) and transfected into OP9 cells using RNAi-Max (Invitrogen) and a reverse-transfection protocol. DNA transfection was carried out by retroviral infection. siRNA specificity was verified using a second diced pool of siRNA, as well as with synthetic siRNA (see Figures S2A–S2C). Sources for antibodies, reagents, constructs, and primers are provided in the Extended Experimental Procedures.

### Automated Image Acquisition and Processing

Images were acquired on an ImageXpress 5000A automated epifluorescence microscope (Molecular Devices, Sunnyvale, CA, USA) using a 4X Plan Fluor objective and a 1,280 × 1,024 pixel, cooled CCD camera with a 12-bit readout. Image analysis was performed using custom software written in MATLAB. In brief, nuclear centroids were identified in images of Hoechst stain. A nucleus mask was generated for each cell by expansion from the centroid to reach 30% of maximum intensity. A cell mask was then generated by expansion of the nucleus mask 7 μm to include both the nucleus and the perinuclear region. After local background subtraction, the nucleus mask was used to measure PPARγ, C/EBPβ, and C/EBPα mean intensities, and the cell mask was used to measure BODIPY (lipid droplet content) and p-AKT mean intensities.

### Computational Model

MATLAB SimBiology was used to program and run the model simulations. The model equations used to generate Figures 5B and 5C are presented below. Additional details can be found in the Extended Experimental Procedures.

$$\begin{array}{c} \frac{\partial \left[CEBP\beta \right]}{\partial t}=synCEBP\beta \;*\;\left(baseCEBP\beta +\left[GR\right]\;*\;\left[cAMP\right]+\frac{{\left[PPAR\gamma \right]}^{2}}{\alpha_{1}^{2}+{\left[PPAR\gamma \right]}^{2}}\right)-\deg CEBP\beta \;*\;\left[CEBP\beta \right]\\ \frac{\partial \left[PPAR\gamma \right]}{\partial t}=synPPAR\gamma \;*\;\left[basePPAR\gamma +\left(\frac{{\left[CEBP\beta +CEBP\alpha \right]}^{3}}{\alpha_{2}^{3}+{\left[CEBP\beta +CEBP\alpha \right]}^{3}}\right)\;*\;\frac{\left[pAKT\right]}{\alpha_{3}+\left[pAKT\right]}\right]-\mathrm{degPPAR}\gamma \;*\;\left[\mathrm{PPAR}\gamma \right]\\ \frac{\partial \left[CEBP\alpha \right]}{\partial t}=synCEBP\alpha \;*\;\left(baseCEBP\alpha +\frac{{\left[PPAR\gamma \right]}^{3}}{\alpha_{4}^{2}+{\left[PPAR\gamma \right]}^{3}}\right)-\deg CEBP\alpha \;*\;\left[CEBP\alpha \right]\\ \frac{\partial \left[pAKT\right]}{\partial t}=synpAKT\;*\;\left(\left(baseIR+\left[IR\right]\right)\;*\;\frac{\alpha_{5}}{\alpha_{5}+\left[cAMP\right]\;*\;\left[GR\right]}\right)-\deg pAKT\;*\;\left[pAKT\right]\\ \frac{\partial \left[IR\right]}{\partial t}=synIR\;*\;\left(baseIR+\frac{\left[CEBP\alpha \right]}{\alpha_{6}+\left[CEBP\alpha \right]}\right)-\deg IR\;*\;\left[IR\right]\\ \frac{\partial \left[Fat\right]}{\partial t}=synFat\;*\;\frac{\left[pAKT\right]}{\alpha_{7}+\left[pAKT\right]}\;*\;\frac{\left[PPAR\gamma \right]}{\alpha_{8}+\left[PPAR\gamma \right]}-\deg Fat\;*\;\left[Fat\right] \end{array}$$

The model has three inputs: [*IR*], [*GR*], and [*cAMP*].

## Supplementary Material

Supp. Figures

Supp. Tables
