# Supplementary material for: Consecutive Positive Feedback Loops Create a Bistable Switch that Controls Preadipocyte-to-Adipocyte Conversion
Source: Cell Rep. Author manuscript; Available in PMC 2016 Jul 25. (PMC4959269; doi:10.1016/j.celrep.2012.08.038)
Supplement: Supp. Figures [file NIHMS741645-supplement-Supp__Figures.pdf]

Cell PRESS Open ACCESS

EXTENDED EXPERIMENTAL PROCEDURES

siRNA Transfection

Synthetic SMARTPOOL siRNA for PPARγ, C/EBPβ, and C/EBPα were purchased from Dharmacon. To generate diced siRNA, gene-specific primers were designed with an in-house primer program and were used to generate ~600 bp cDNA fragments immediately upstream of the stop codon of each mRNA by PCR. An additional set of nested primers was designed to add T7 promoters at both ends of the final cDNA fragment. Nested PCR products were subjected to in vitro transcription, in vitro dicing, and purification to produce siRNA as described previously (Galvez et al., 2007). The OP9 cells were transfected with siRNA by a reverse transfection protocol. For each 96-well well, 2 pmol of diced-pool siRNA was diluted in 10ul of Opti-Mem I Medium. 0.2ul of RNAiMax (Invitrogen) diluted in 10ul of Opti-Mem I was then added, mixed well, and then incubated for 20 min at room temperature. This mixture was then placed into a 96-well, and OP9 cells were added (15,000 cells suspended in 80 μl of growth medium without antibiotics). After 24 hr, the media was replaced with differentiation media to induce differentiation following differentiation method. Specificity for all siRNA used in the paper - PPARγ, C/EBPβ, C/EBPα, glucocorticoid receptor, and insulin receptor – was verified by demonstrating that the same results were obtained using two different diced pools of siRNA each targeting a different coding region of the respective gene (Figures S2B and S2C). Specificity of PPARγ, C/EBPβ, C/EBPα siRNA was also confirmed using commercially-obtained synthetic siRNA.

Retroviral Infection of Cells

HEK293T were transfected in 6-well wells at 30% confluence using Fugene6 and 1 μg of PCL-ECO packaging construct together with either 1 μg of pBMNi-PPARγ-IRES-hcRed, pBMNi-C/EBPα-IRES-GFP, or pBMNi-IRES-GFP expression plasmids. 4 ml of viral supernatant was collected both at 48 and 72 hr after transfection. The 8 ml total of viral supernatant was combined, concentrated using PEG-it virus precipitation solution (System Biosciences), and resuspended in 100ul of PBS to make concentrated retrovirus. 5000 OP9 cells in 100ul of OP9 growth media were plated into each 96-well well 12 hr before infection. 5 ul of concentrated retrovirus plus polybrene (8mg/ml final concentration) was added per 96-well well. After 12 hr, the media in each well was changed to fresh OP9 growth media. Cells were fixed 10 days after infection.

Immunofluorescence Staining

OP9 cells were fixed with 3% paraformaldehyde in PBS for 30 min. Then the cells were gently washed 3X with PBS and permeabilized with 0.05% saponin (Sigma #47036), blocked with 3% bovine serum albumin (Sigma #7906) and stained with anti-PPARγ (1:500 Santa Cruz Biotech #sc-7273), anti-C/EBPα (1:500, Santa Cruz Biotech #sc-61), anti-C/EBPβ (1:500, Santa Cruz Biotech #sc-150), anti-C/EBPβ (1:500, Santa Cruz Biotech #sc-7962), anti-pAKT(S473)(1:1000, Cell Signaling #4060) and BODIPY 493/503 (1ug/ml, Molecular Probes #D-3922). Alexa Fluor-514 (#A31558), 555 (#A21429), 594 (#A11032) and 647 (#A31571) (1:1000, Invitrogen) were used as secondary antibodies.

Western Blot Analysis

Cultured cells were lysed using the NE-PER nuclear and Cytoplasmic extraction kit (Pierce; Rockford, IL) for nuclear and cytoplasmic lysate. Protein concentrations were determined by BCA protein assay kit (Pierce) with BSA as standard. Gel electrophoresis was performed using precast Bis-Tris 4%–12% gradient polyacrylamide gels with NuPAGE buffer system (Invitrogen). Proteins were transferred to PVDF membranes (Immobilon-FL,Millipore) using the Xcell II Blot Module (Invitrogen). The membranes were blocked for 1 hr in Odyssey blocking buffer (Li-Cor biosciences, Cambridge, UK) and were probed with the following primary antibodies: PPARγ, C/EBPα, C/EBPβ and Insulin receptorβ (1:500, Santa Cruz Biotechnology, Inc.), aP2/FAPB4 (1:3000, Cell Signaling Technology), adiponectin and perilipin (1:1000, Abcam). Detection was performed using a secondary antibody (1:3000) coupled to an IR 680 or IR 800 dye, and the membranes scanned using the Li-Cor Odyssey IR imager.

Model Description

$$\frac{\partial[CEBP\beta]}{\partial t} = synCEBP\beta * \left(baseCEBP\beta + [GR]*[cAMP] + \frac{[PPAR\gamma]^2}{\alpha_1^2 + [PPAR\gamma]^2}\right) - \mathrm{deg}CEBP\beta * [CEBP\beta]$$

$$\frac{\partial[PPAR\gamma]}{\partial t} = synPPAR\gamma * \left[basePPAR\gamma + \left(\frac{[CEBP\beta + CEBP\alpha]^3}{\alpha_2^3 + [CEBP\beta + CEBP\alpha]^3}\right) * \frac{[pAKT]}{\alpha_3 + [pAKT]}\right] - \mathrm{degPPAR}\gamma * [\mathrm{PPAR}\gamma]$$

$$\frac{\partial[CEBP\alpha]}{\partial t} = synCEBP\alpha * \left(baseCEBP\alpha + \frac{[PPAR\gamma]^3}{\alpha_4^2 + [PPAR\gamma]^3}\right) - \mathrm{deg}CEBP\alpha * [CEBP\alpha]$$

$$\frac{\partial[pAKT]}{\partial t} = synpAKT * \left((baseIR + [IR]) * \frac{\alpha_5}{\alpha_5 + [cAMP] * [GR]}\right) - \text{deg}pAKT * [pAKT]$$

$$\frac{\partial[IR]}{\partial t} = synIR * \left(baseIR + \frac{[CEBP\alpha]}{\alpha_6 + [CEBP\alpha]}\right) - \text{deg}IR * [IR]$$

$$\frac{\partial[Fat]}{\partial t} = synFat * \frac{[pAKT]}{\alpha_7 + [pAKT]} * \frac{[PPAR\gamma]}{\alpha_8 + [PPAR\gamma]} - \text{deg}Fat * [Fat]$$

The fixed parameters used were: degCEBPB = 0.1 (rel unit), degPPARG = 0.15, degCEBPA = 0.2, degFAT = 0.06, degpAKT = 0.5, synCEBP = 0.075, synPPARG = 0.7, synCEBPA = 0.7, synFAT = 0.25, synpAKT = 0.08, alpha1 = 1.0, alpha2 = 1.0, alpha3 = 1.0, alpha4 = 1.5, alpha5 = 0.5, BasePPARG = 0.06, BaseCEBPA = 0.06, BaseCEBPB = 0.05, alpha6 = 0.6, alpha7 = 0.1, BaseIR = 0.005, alpha8 = 1.0, synIR = 0.6, degIR = 0.05.

Initial values for the three inputs: insulin, [IR] = 0.25 (rel. unit); [cAMP] = 2.0; glucocorticoids. [GR] = 2.0.

Specific details of each equation:

(1) d[CEBPβ]/dt: The addition of GR and cAMP has been shown to directly cause upregulation of C/EBPβ expression. The PPARγ term reflects the fact that C/EBPβ is in a positive feedback loop with PPARγ. In addition to the experimental results we present in this paper, the existence of a feedback loop between PPARγ to C/EBPβ is supported by the fact that C/EBPβ has been shown to bind directly to the PPARg promoter (Schmidt et al., 2011) and PPARγ to the C/EBPβ promoter (Mikkelsen et al., 2010). However, despite the acquisition of several chromatin immunoprecipitation sequencing data sets (Siersbæk et al., 2011; Steger et al., 2010; Schmidt et al., 2011), there is no evidence that C/EBPβ binds to the C/EBPα promoter and vice versa. Thus, it is more likely that C/EBPβ is in direct feedback loop with PPARγ but not with C/EBPα.

(2) d[PPARγ]/dt: Because they can bind to the same DNA sequences and can replace each other at binding sites, C/EBPα and C/EBPβ are added together in the term reflecting the positive feedback between these transcription factors (TFs) and PPARγ. The pAKT term reflects the positive feedback between the insulin signaling pathway and PPARγ. There is no Hill coefficient in the pAKT feedback because our experiments show that pAKT signaling is always graded and never bimodal.

(3) There are two main mechanisms of cooperativity in this system: 1) C/EBPβ and C/EBPα need to dimerize to activate transcription and 2) there are multiple binding sites for C/EBPα and C/EBPβ on the PPARγ promoter and multiple binding sites for PPARγ on the C/EBPβ and C/EBPα promoters (Lefterova et al., 2008; Mikkelsen et al., 2010; Steger and Lazar, 2011). Thus Hill coefficients of at least 2 were chosen for the feedback loops in the first 3 equations. Given that the precise cooperativity is not known and is difficult to measure experimentally, we chose the respective Hill coefficients because lower cooperativity did not give sufficient bistability to match the experimental data. In the model, we used the minimal cooperativity needed to recapitulate the experimental data.

(4) d[pAKT]/dt: pAKT is a readout of the insulin pathway activity. pAKT is inhibited by [cAMP] and [GR] stimulation.

(5) d[IR]/dt: C/EBPα upregulates IR expression.

(6) d[Fat]/dt: The amount of fat is dependent on both AKT activity (pAKT) and PPARγ expression. However, the constants alpha7 and alpha8 were shown to reflect that the amount of fat is much more strongly dependent on pAKT than on PPARγ expression.

(7) Both PPARγ and C/EBPα bind to their own promoters respectively (Lefterova et al., 2008; Steger et al., 2010) which suggests that these transcription factors could be regulated by autofeedback loops. We did not include autofeedback for PPARγ or C/EBPα because there is no experimental evidence yet in the literature that they are regulated by autofeedback. Even though binding sites are present, one cannot assume actual transcriptional regulation without testing for this. An additional reason why we did not include autofeedback for PPARγ is that our experimental results (Figure 3E) showed that knockdown of C/EBPα and subsequent activation of PPARγ resulted in an almost complete reduction of PPARγ expression, indicating at best only a minor role of an autofeedback PPARγ loop if it does prove to exist. We did not include any autofeedback term in the C/EBPβ equation in the model since no evidence has been shown yet even in large-scale CHIP studies that C/EBPβ binds to its own promoter (Siersbæk et al., 2011; Steger et al., 2010).

(8) Small random variations were introduced to model parameters assuming log normal distribution of the parameters.

(9) All models presented in this manuscript will be uploaded to the EMBL-EBI BioModels repository: (http://www.ebi.ac.uk/biomodels-main/).

SUPPLEMENTAL REFERENCES

Galvez, T., Teruel, M.N., Heo, W.D., Jones, J.T., Kim, M.L., Liou, J., Myers, J.W., and Meyer, T. (2007). siRNA screen of the human signaling proteome identifies the PtdIns(3,4,5)P3-mTOR signaling pathway as a primary regulator of transferrin uptake. Genome Biol. *8*, R142.

Lefterova, M.I., Zhang, Y., Steger, D.J., Schupp, M., Schug, J., Cristancho, A., Feng, D., Zhuo, D., Stoeckert, C.J., Jr., Liu, X.S., and Lazar, M.A. (2008). PPAR-gamma and C/EBP factors orchestrate adipocyte biology via adjacent binding on a genome-wide scale. Genes Dev. *22*, 2941–2952.

Mikkelsen, T.S., Xu, Z., Zhang, X., Wang, L., Gimble, J.M., Lander, E.S., and Rosen, E.D. (2010). Comparative epigenomic analysis of murine and human adipogenesis. Cell *143*, 156–169.

Schmidt, S.F., Jørgensen, M., Chen, Y., Nielsen, R., Sandelin, A., and Mandrup, S. (2011). Cross species comparison of C/EBPα and PPARγ profiles in mouse and human adipocytes reveals interdependent retention of binding sites. BMC Genomics *12*, 152.

Siersbæk, R., Nielsen, R., John, S., Sung, M.H., Baek, S., Loft, A., Hager, G.L., and Mandrup, S. (2011). Extensive chromatin remodelling and establishment of transcription factor 'hotspots' during early adipogenesis. EMBO J. *30*, 1459–1472.

Steger, D.J., and Lazar, M.A. (2011). Adipogenic hotspots: where the action is. EMBO J. *30*, 1418–1419.

Steger, D.J., Grant, G.R., Schupp, M., Tomaru, T., Lefterova, M.I., Schug, J., Manduchi, E., Stoeckert, C.J., Jr., and Lazar, M.A. (2010). Propagation of adipogenic signals through an epigenomic transition state. Genes Dev. *24*, 1035–1044.

Cell PRESS
Open ACCESS

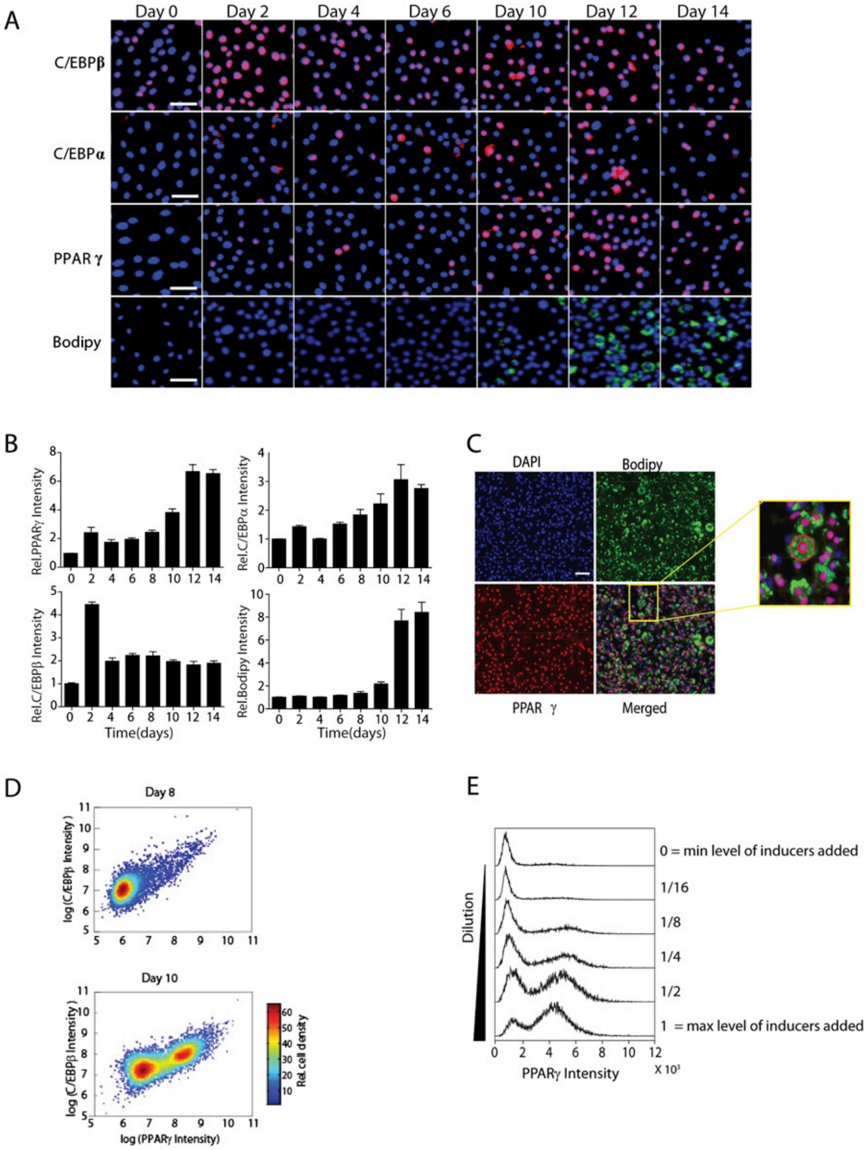

Figure S1. Development of a Single-Cell Assay to Quantitatively Measure Key Adipogenic Parameters in OP9 and 3T3-L1 cells, Related to Figure 1

(A and B) The changes in the concentration of key transcription factors and lipid content (BODIPY) during adipogenesis occur in the same sequence in 3T3-L1 cells as in OP9 cells (Figure 1). (A) 3T3-L1 cells were induced to differentiate using the protocol described in the Experimental Procedures section. Immunohistochemistry staining of 3T3L1 cells using specific antibodies to visualize PPARγ, C/EBPα, and C/EBPβ (red), BODIPY 493/503 to visualize lipid droplets (green), and Hoechst to visualize nuclei (blue). Scale bar, 40 μm. (B) PPARγ, C/EBPα and C/EBPβ concentrations obtained by averaging intensities of antibody staining from the nuclei of individual 3T3-L1 cells. Total cellular lipid droplet content obtained by averaging BODIPY intensities from the cytosol of individual cells. Approximately 30,000 cells were used for each time point. Error bars show standard error (mean ± SD of four replicate wells). All values were normalized to the unstimulated (Day 0) level of each value.

(C) High-content, image-based, multi-parameter, single-cell analysis of fat cells. OP9 cells were co-stained with a specific antibody to PPARγ (red), BODIPY 493/503 to visualize lipid droplets (green), and Hoechst to visualize nuclei (blue). Scale bar, 50 μm.

(D) Higher levels of differentiation inducers results in more uniform differentiation. Histograms showing the number of cells (*y* axis) with the specified concentrations of PPARγ (*x* axis) 96 hr after the induction of differentiation. Two-fold serial dilutions of inducers were added to the medium of undifferentiated OP9 cells to initiate differentiation. The medium was replaced 48 hr later with medium containing 175 nM insulin. The maximum level of inducers added (corresponding to a value of "1") was 175 nM insulin, 0.5 mM 3-isobuytl-1-methylxanthine, and 1μM dexamethasone. Approximately 25,000 cells were used for each histogram.

(E) 3T3-L1 cells also show bimodality during differentiation. Single-cell analysis of the 3T3-L1 cells imaged in Figure S1A.

Cell PRESS
Open ACCESS

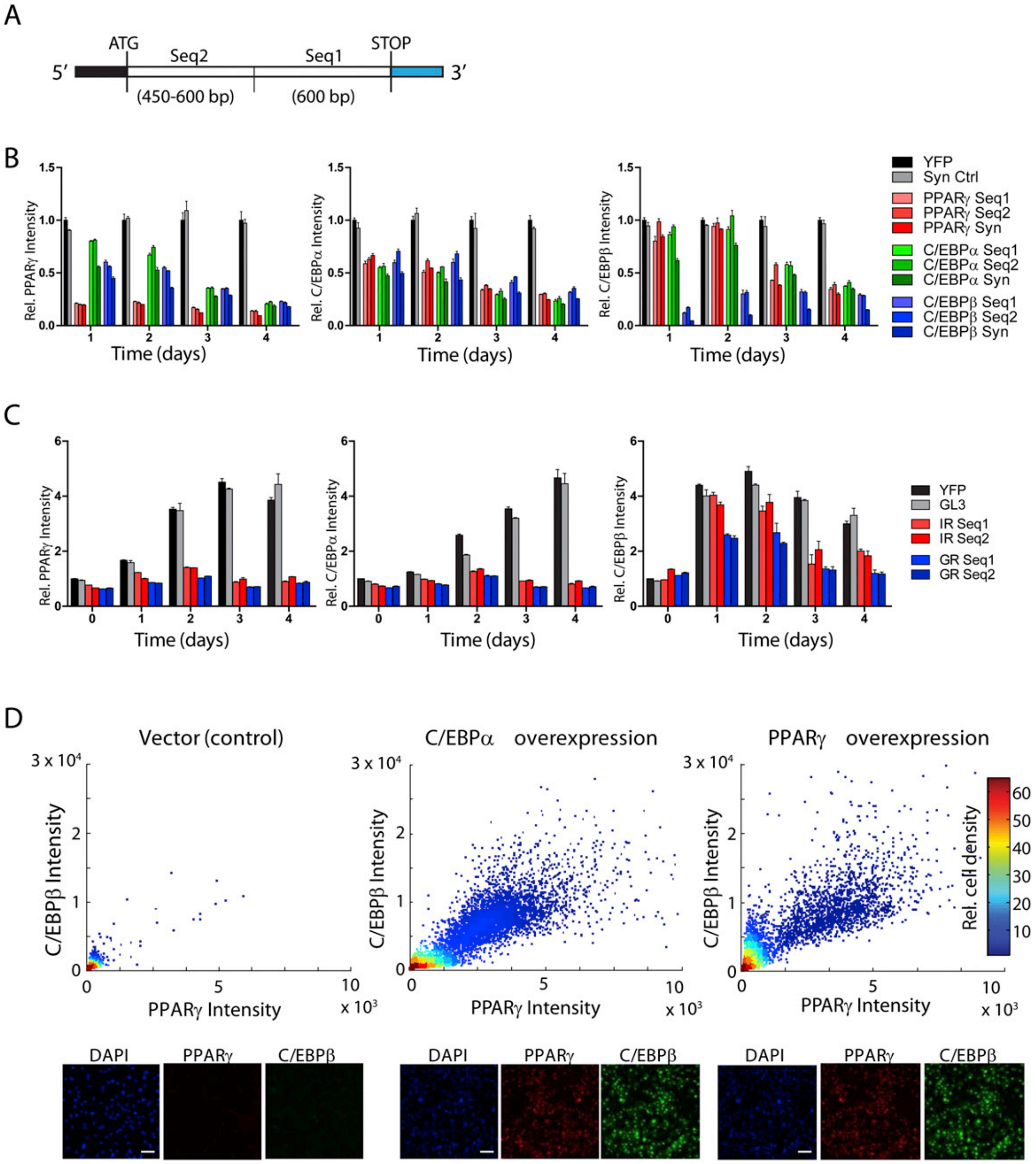

Figure S2. Verifying siRNA Specificity and the Existence of a 3T3-L1 PPARγ-C/EBPβ Feedback Loop, Related to Figure 2

(A) To generate two independent diced siRNA pools (d-siRNA) for each gene, primers were designed to amplify two different coding regions of each gene (Seq1 and Seq2). Tables S1 and S2 show the primers used to make the Seq1 and Seq2 d-siRNA for each gene.

(B and C) Similar to the experiments presented in Figures 2B and 4B, OP9 cells were transfected with d-siRNA or synthetic siRNA (20nM) and 24 hr later were stimulated to differentiate with insulin, glucocorticoid, and cAMP stimuli. Cells were fixed at the respective time points, stained with antibodies to PPARγ, C/EBPα and C/EBPβ, and analyzed by epifluorescence microscopy. Each bar represents the average of approximately 25,000 cells (mean ± SD of three replicate wells). All values were normalized to the average intensity of the YFP d-siRNA transfected cells value at each time point (B) or to the average intensity of the YFP d-siRNA transfected cells at Day 0 (C).

(D) The feedback loop from PPARγ to C/EBPβ also exists in 3T3-L1 cells. Overexpression of PPARγ and C/EBPα using retroviruses. Retrovirus infected 3T3-L1 cells with control pBMNi-GFP vector or pBMNi-C/EBPα-GFP or pBMNi- PPARγ-hcRed construct were fixed at day 10. Cells were stained with specific antibody to PPARγ and C/EBPα or PPARγ and C/EBPβ and analyzed by epifluorescence microscopy. Scale bar, 50 μm.

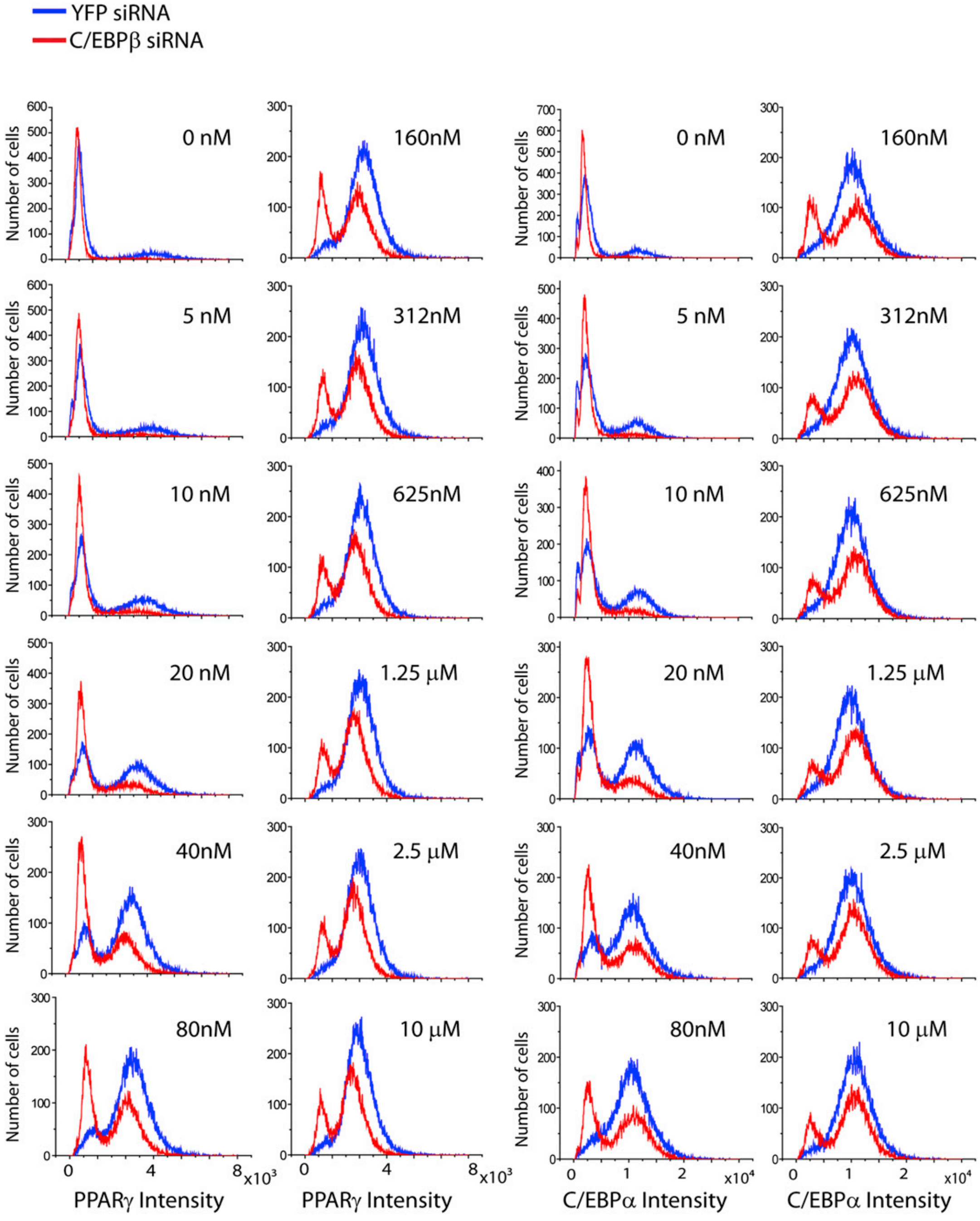

Figure S3. The PPARγ-C/EBPβ Feedback Loop Makes the Differentiation Switch More Robust, Related to Figure 3

If the PPARγ-C/EBPβ feedback loop is suppressed by siRNA knockdown of C/EBPβ (red curves), even maximal doses of rosiglitazone cannot convert all cells into the differentiated, high PPARγ state as is the case with control cells transfected with YFP siRNA (blue curves) Each histogram represents PPARγ and C/EBPα nuclear intensities from approximately 30,000 cells measured with immunohistochemistry and epifluorescence imaging. Undifferentiated OP9 cells were stimulated with different doses of rosiglitazone (from 5nM to 10 μM). Cells were fixed and stained with PPARγ and C/EBPα antibodies 48 hr later.

Cell PRESS
Open ACCESS

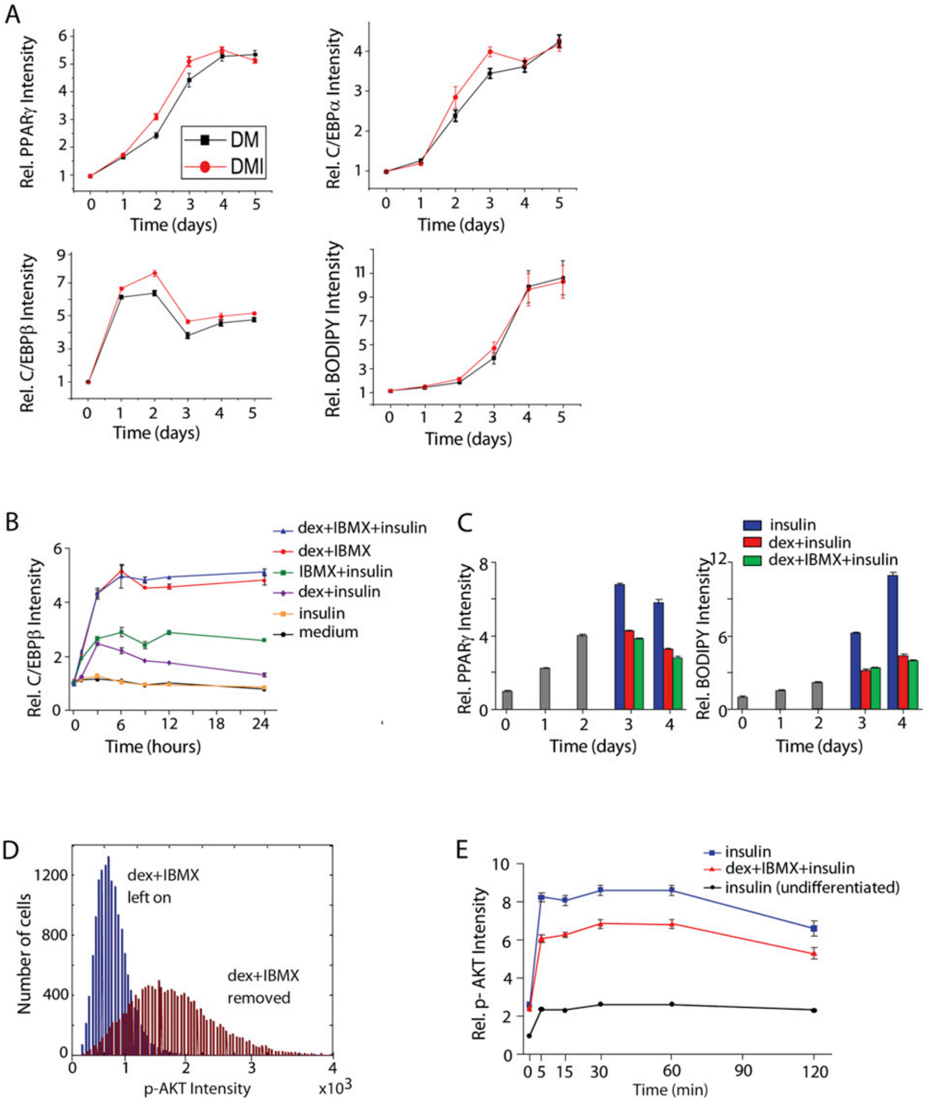

Figure S4. Elucidation of the Role of the Insulin Pathway in Adipogenesis, Related to Figure 4

(A) Insulin has minimal effect on differentiation during the first 2 days. Insulin+IBMX+dex versus IBMX+dex time courses of PPARγ, C/EBPα, C/EBPβ expression and lipid content. OP9 cells were induced to differentiate by exposure to DM (Dex+IBMX) or DIM (DM with Insulin). After 2 days, the medium was replaced with medium containing 175nM insulin, and the cells were fixed at the indicated time points. Cells were stained with PPARγ, C/EBPα, C/EBPβ antibodies and BODIPY 493/503 for lipid contents measurement All values are normalized to the unstimulated (Day 0) level of each value. Approximately 30,000 cells were used for each data point (mean ± SD of four replicate wells).

(B–E) cAMP and glucocorticoids are needed early in differentiation, but later are later inhibitory via suppression of insulin signaling. (B) Plot of C/EBPβ expression over time in response to different combinations of adipogenic inducers. Approximately 30,000 cells were used for each data point (mean ± SD of four replicate wells). cAMP and glucocorticoid receptors were required to increase C/EBPβ expression for a 48 hr period, triggering PPARγ and C/EBPα induction which then in turn further amplified C/EBPβ. Insulin was not needed for C/EBPβ expression, as was shown by the fact that dex+IBMX addition with or without insulin resulted in the same amount of C/EBPβ expression. Dex (dexamethasone) is a synthetic glucocorticoid. IBMX raises cAMP levels in cells. (C) dex+IBMX reduces PPARγ expression and lipogenesis by over 50 percent if left in the medium past 48 hr. Time course of PPARγ expression and lipid droplet accumulation in OP9 cells that were induced to differentiate by the addition of medium (DIM) containing dex (1 μM), IBMX (500nM), insulin (175nM), and 10% FBS for 48 hr. The medium was then replaced with medium containing the indicated combinations of the adipogenic inducers. Cells were fixed and stained at the respective time points. Approximately 30,000 cells were used for each bar (mean ± SD of four replicate wells). (D) By monitoring phosphorylation of Akt, we found that cAMP and glucocorticoid blocked p-AKT and thus the insulin signaling pathway. These histograms show the number of cells with the specified concentrations of p-AKT 72 hr after the induction of adipogenesis by addition of dex+IBMX+insulin when dex + IBMX was removed at 48 hr or left on. Approximately 7000 cells were plotted for each condition. (E) When we removed IBMX+dex after 48 hr and monitored p-AKT expression, p-AKT signaling was restored within 5 min, indicating that the block on p-AKT was at least partially due to a rapid signaling block as shown by this time course of p-AKT expression obtained immediately after the removal of IBMX+dex. Cells were differentiated with IBMX+dex for 48 hr, and then the medium was changed to medium containing either insulin or DIM. For all panels, OP9 cells were fixed at the indicated time points, stained with the respective antibodies, and then imaged to obtain fluorescence intensities. All values are normalized to the basal level (unstimulated) of each values. Approximately 30,000 cells were used for each data point (mean ± SD of four replicate wells).

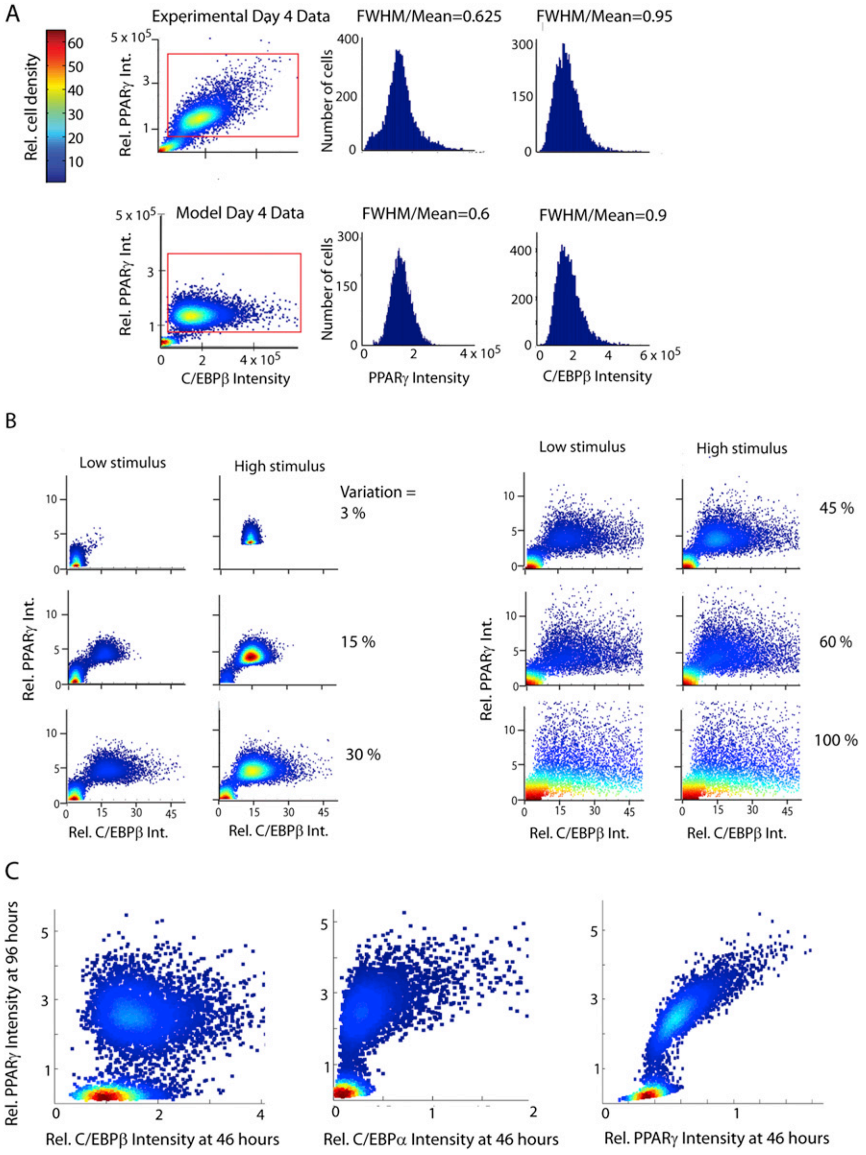

Figure S5. Role of Variations in Cell-to-Cell Protein Expression in Controlling the Number of Differentiated Fat Cells, Related to Figure 5

(A) The top plots show single-cell immunohistochemistry data obtained from ~10,000 individual OP9 cells at Day 4 of differentiation induced by addition of the adipogenic cocktail. This is the same data shown in Figure 5C, but now plotted as a 2D density scatterplot. The 1D histograms show the distribution of intensities of PPARγ and C/EBPβ in the high PPARγ / high C/EBPβ peaks (shown in the red boxes). To obtain an estimate of how much variation there is in the synthesis and degradation rates of PPARγ, C/EBPβ, and C/EBPα, different amounts of variation were introduced into the model until the computationally-obtained FWHM/Mean values of the histograms for PPARγ and C/EBPβ – after running the model 10,000 times to simulate 10,000 individual cells - matched the experimentally-obtained FWHM/Mean values. The best match between the experimental and computational FWHM/Mean values was obtained when on average the synthesis and degradation rates, as well as the basal values, of PPARγ were varied by 15% and of C/EBPβ and C/EBPα were varied by 30%.

(B) With too little or too much protein variation, the bistable switch breaks down. Each row shows results of simulations with increasing variation in the synthesis and degradation of C/EBPα, C/EBPβ, and PPARγ, with low stimulation (GR = cAMP = 0.3 rel units) being applied in the left plots and high stimulation (GR = cAMP = 2.0 rel units) in the right plots. Each plot shows the results of 10,000 stimulations. With just 3% variation, either all the cells switch or all the cells remain undifferentiated for a given submaximal stimulus. With increasing variation, two populations are evident and increasing the stimulus just switches more cells from the low PPARγ population to the high PPARγ population. With too much variation (for example, 100%), the bimodality breaks down and there is no longer 2 distinct populations. Because it was found in Figure S5A that the synthesis and degradation rates as well as the basal levels, of PPARγ, needed to vary about half the amount of those of C/EBPβ and C/EBPα in order to match experimental data, in the data presented in here in Figure S5B as well as S5C, the variation in PPARγ was always introduced as half the variation in C/EBPβ and C/EBPα parameters. In other words, when the figure text says a "Variation of 3%," that means that the C/EBPα and C/EBPβ parameters were varies by 3% and the PPARγ parameters were varied by only 1.5%.

(C) Modeling results comparing PPARγ expression at Day 4 with expression of C/EBPα, C/EBPβ, and PPARγ expression 46 hr after induction of differentiation by addition of cAMP and glucocorticoids. The plots show the results of 10,000 stimulations. For each simulation, the synthesis and degradation rates, as well as the basal levels, were randomly varied on average 30% for C/EBPβ and C/EBPα and 15% for PPARγ.

Cell PRESS
Open ACCESS

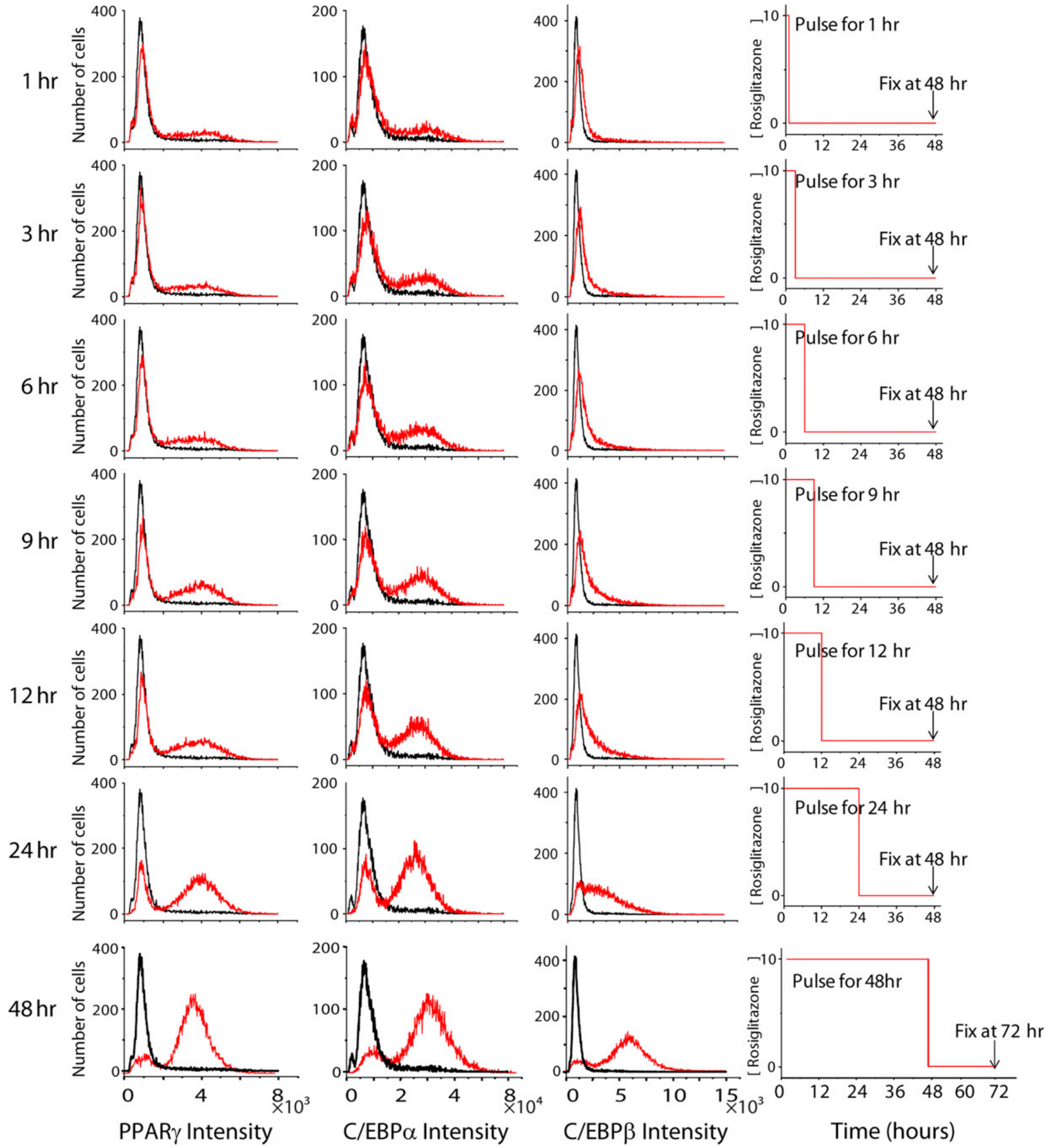

Figure S6. Increasing the Activation Pulse Time Locks More and More Cells into a Terminal Differentiation State, Related to Figure 7

Each histogram represents PPARγ, C/EBPα and C/EBPβ nuclear intensities from approximately 30,000 cells. At time 0, undifferentiated OP9 cells were stimulated with rosiglitazone (10 μM), red curves) for different pulse time periods or left in basal media (black curves), then washed three times with fresh medium, and then placed in fresh medium without rosiglitazone. Cells were fixed, stained with the respective antibodies, and analyzed using epifluorescence imaging.
