# Supplementary material for: Consecutive Positive Feedback Loops Create a Bistable Switch that Controls Preadipocyte-to-Adipocyte Conversion
Source: Cell Rep. Author manuscript; Available in PMC 2016 Jul 25. (PMC4959269; doi:10.1016/j.celrep.2012.08.038)
Supplement: Supp. Tables [file NIHMS741645-supplement-Supp__Tables.pdf]

Table S1: Primers used to make diced siRNA pools (d-siRNA) targeting the Seq1 sequence of the respective gene product. This Seq1 d-siRNA was used to generate Figures 2B and 4B.

Gene name	Protein Accession	First primer Forward	First primer Reverse	Nested primer Forward	Nested primer reverse
**Pparg**	NP_035276	TCCGAATTTTTCAAGGGTGCC	CAAGTCCTTGTAGATCTCCTG	GCGTAATACGACTCACTATAGGGTTTCGATCCGTAGAAGCC	GCGTAATACGACTCACTATAGGGTGAAGGCTCATGTCTGTC
**Cebpa**	NP_031704	CTCTTCCCCTACCAGCCAC	CTTGACCAAGGAGCTCTCAG	GCGTAATACGACTCACTATAGGCACCCGCACGCGTCTC	GCGTAATACGACTCACTATAGGGCAGCGTGTCCAGTTCAC
**Cebpb**	NP_034013	CATGGCGGCCGGTTTCC	GGCAGCTGCTTGAACAAGTTC	GCGTAATACGACTCACTATAGGGTTTCCCGTTCGCCCTG	GCGTAATACGACTCACTATAGGAGCTGCTCCACCTTCTTC
**Insr**	NP_03498	CAGATTACTATCGGAAAGGGG	TTAGGAAGGGTTTGACCTTGG	GCGTAATACGACTCACTATAGGTTCCTGTGAGGTGGATGTC	GCGTAATACGACTCACTATAGGAAGGACACGTCCGTTCTTC
**Nr3c1**	NP_032199	GCGATACCAGGATTCAGAAAC	TCTGATGAAACAGAAGCTTTTTG	GCGTAATACGACTCACTATAGGCCTGGATGACCAAATGACC	GCGTAATACGACTCACTATAGGCTGATTAGTGATGATTTCAGC
**YFP**				GCGTAATACGACTCACTATAGGCATCCTGGTCGAGCTGGAC	GCGTAATACGACTCACTATAGGCGTTGGGGTCTTTGCTCAG
**GL3**				GCGTAATACGACTCACTATAGGGCGGTCGGTAAAGTTGTTC	GCGTAATACGACTCACTATAGGTCTTGCGTCGAGTTTTCCG

Table S2: Primers used to make d-siRNA targeting the Seq2 sequence of the respective gene product.

Gene name	Protein Accession	First primer Forward	First primer Reverse	Nested primer Forward	Nested primer reverse
**Pparg**	NP_035276	AAGTAGAACCTGCATCTCCAC	TGCAGGGGGGTGATATGTTTG	GCGTAATACGACTCACTATAGGAGACCCAGCTCTACAACAG	GCGTAATACGACTCACTATAGGATCTTCTCCCATCATTAAGG
**Cebpa**	NP_031704	GAGTCGGCCGACTTCTACG	TGTTTGATCACCAGCGGCCG	GCGTAATACGACTCACTATAGGCTTCTACGAGGTGGAGCCG	GCGTAATACGACTCACTATAGGTCGTACAGGGGCTCCAG
**Cebpb**	NP_034013	CACCGCCTGCTGGCCTG	GTCCGCGCGCTTGCAGTC	GCGTAATACGACTCACTATAGGGCCTTTAGACCCATGGAAGTG	GCGTAATACGACTCACTATAGGGTTCGAAGCCCGGCTCCG
**Insr**	NP_03498	AATGGGACCACTGTATGCATC	GTCCCTTGTCATTCCAAAGTC	GCGTAATACGACTCACTATAGGCAAACCCTGAGTACCTCAG	GCGTAATACGACTCACTATAGGATCATGGGCAACCATGCAG
**Nr3c1**	NP_032199	TCCTTGGGGGCTATGAACTTC	CCATTTCACTGCGGCAATCAC	GCGTAATACGACTCACTATAGGATGGATATTCAAGCCCTGG	GCGTAATACGACTCACTATAGGGCCCACCTAACATGTTGAG
